# From community engagement to lived experience leadership: a systematic review of HIV services among men who have sex with men

**DOI:** 10.1093/heapro/daag084

**Published:** 2026-07-02

**Authors:** Aron Harold G Pamoso, Elizabeth Waldron, I Nyoman Sutarsa, Mary Lou Rasmussen, Brett Scholz

**Affiliations:** School of Medicine and Psychology, The Australian National University, Building 39, 39 Science Road, Canberra ACT 2601,Australia; School of Medicine and Psychology, The Australian National University, Building 39, 39 Science Road, Canberra ACT 2601,Australia; School of Medicine and Psychology, The Australian National University, Building 39, 39 Science Road, Canberra ACT 2601,Australia; School of Sociology, Research School of Social Sciences, The Australian National University, 146Ellery Crescent, Canberra ACT 2601, Australia; School of Medicine and Psychology, The Australian National University, Building 39, 39 Science Road, Canberra ACT 2601,Australia

**Keywords:** men who have sex with men (MSM), lived experience leadership, community engagement, HIV health services, systematic review, intersectionality, health equity, health policy

## Abstract

Community responses are essential to achieving the global goal of Ending AIDS by 2030, yet men who have sex with men (MSM) remain underrepresented and often tokenized within HIV initiatives. While community engagement is widely emphasized, far less attention has been given to leadership rooted in lived experience. Meaningful community engagement requires recognizing MSM not merely as a target population, but as leaders shaping HIV responses. This systematic review examines how MSM perceive the use of their lived experiences in HIV responses and how institutions incorporate these experiences into service delivery. Following PRISMA guidelines, we analyzed 40 peer-reviewed studies. Results show that across diverse contexts, MSM navigate systemic barriers—including intersecting systems of oppression—that hinder equitable access to HIV care. Despite these challenges, MSM assume (in)formal roles of leadership, negotiating power, and agency within institutions that have historically marginalized them. Using lived experience leadership as a framework in HIV services characterizes meaningful community engagement among MSM and their communities, including pathways for strengthening community-engaged HIV health promotion. This synthesis underscores the need to move beyond narrow notions of community engagement and toward recognizing MSM as leaders whose experiential expertise can enhance equity, relevance, and effectiveness in HIV health promotion programs and provides actionable insights for community-engaged service design and policy.

Contribution to Health Promotion

- Many HIV programs continue to tokenize men who have sex with men (MSM), limiting their meaningful engagement in service delivery.
- Lived experience leadership provides a framework that positions MSM as experts who actively shape and transform HIV health promotion programs.
- Integrating MSM’s experiential expertise can improve cultural alignment, enhance equity, and strengthen the quality and relevance of services, offering guidance for designing inclusive, lived experience-led interventions.
- Implementing lived experience leadership through an intersectional framework in HIV services, policy, education, and evaluation can contribute to more authentic involvement and representation of MSM and their communities.

## Introduction

Community-engaged HIV responses are central to efforts to address ongoing HIV epidemics and achieve the global target of “Ending AIDS by 2030” (UNAIDS 2021). These responses are designed, implemented, and led by communities themselves, aiming to improve health outcomes, uphold human rights, and ensure services are culturally relevant. UNAIDS (2021) recommends that at least 30% of HIV-related services be community-engaged, with 6% of resources allocated to social enablers, including advocacy, stigma reduction, and policy reform. Across diverse settings, community-driven initiatives enhance the reach, relevance, and effectiveness of HIV prevention, treatment, and care, and have contributed to policy and practice reforms at local and national levels (Ayala *et al*. 2021, UNAIDS 2021).

Despite these commitments, many communities remain marginalized in shaping HIV services (Matlapeng *et al*. 2023). Health systems often operate hierarchically, limiting meaningful community involvement (Andrasik *et al*. 2014). In particular, MSM—a key population affected by HIV—are frequently involved tokenistically, with limited decision-making power (Quinn *et al*. 2020). Such engagement risks undermining the movement toward lived experience leadership, which emphasizes the authentic inclusion of experiential expertise in policy development, program design, service delivery, and evaluation (Scholz *et al.* 2024b).

The concept of lived experience leadership has been more clearly articulated and expanded upon within mental health consumer movements, which involves incorporating the shared expertise of individuals and communities into health service decision-making (Stewart *et al*. 2019). While much literature has focused on mental health, the principles are increasingly relevant in HIV responses. Involving MSM as leaders allows HIV services to draw on experiential knowledge, improve cultural alignment among MSM and HIV services, and meaningful representation in the HIV care continuum (Pamoso *et al*. 2025a). However, most studies we reviewed did not explicitly use the term lived experience leadership, suggesting that involvement with patients and communities may not necessarily equate to or entail shared leadership. Yet, this shift reflects the emergence of frameworks that guide research and practice grounded in the evaluation of lived experiences (Scholz *et al*. 2024a, 2024b).

The benefits of genuine lived experience involvement are well documented: experiential knowledge enhances service relevance, promotes equity, and fosters empowerment (Happell and Scholz 2018). Superficial community engagement, however, risks tokenism, even with good intentions, and can reinforce existing power imbalances between MSM communities and health systems (Coren *et al*. 2021, Scholz 2022). Addressing these imbalances requires critical attention to how intersecting social identities shape MSM experiences of HIV services.

Intersectionality provides a valuable framework for examining these dynamics. Intersectionality explores how multiple social identities interact with systems of power, shaping inequities, and opportunities (Crenshaw 1989, Collins 2000). Applied to HIV responses, it reveals how stigma, discrimination, and structural oppression related to sexuality, race, class, migration, and HIV status intersect to affect MSM health experiences (Bowleg 2008, 2012, Bowleg *et al*. 2022, Dale *et al*. 2022, Pamoso *et al*. 2025b). Intersectionality also highlights resilience, agency, and leadership within MSM communities, demonstrating how individuals leverage their identities to advocate for themselves and others, foster social change, and support peers (Quinn *et al*. 2022).

Despite the promise of intersectionality in advancing lived experience leadership in HIV responses, little to no review studies have explored how MSM and their communities perceive the use of their lived experiences in informing HIV services. Furthermore, limited attention has been paid to how institutions embed lived experience leadership in their HIV responses. Understanding these processes is essential to designing inclusive, equitable, and culturally aligned HIV programs.

### Study aims

While extant literature has mapped out various community-engaged HIV responses (Trapence *et al*. 2012, Ayala *et al*. 2021, Krulic *et al*. 2022), much of the evidence synthesis has parsed the benefits of peer-and community-engaged interventions in higher uptake of HIV services (Ayala *et al*. 2021), focused on how formalized peer navigation benefits the HIV care continuum for key populations living with HIV (Krulic *et al*. 2022), and interventions tackling intersectional stigma in HIV contexts (Stangl *et al*. 2023). However, existing synthesis of evidence has not taken into consideration how community HIV responses emerged from informal roles, including how key populations such as MSM perceive the involvement of their lived experiences in shaping various HIV services, including limited in-depth engagements of how lived experiences shape HIV services (Stangl *et al*. 2023).

To support the global goal of “Ending AIDS by 2030,” community HIV responses must center the experiential expertise of people and communities at risk and or living with HIV. This ensures equitable, culturally responsive services. This systematic review with narrative synthesis aims to:

1. Explore MSM attitudes toward how their lived experiences inform HIV health service provision.
2. Examine how institutions (e.g. hospitals, clinics, community-based organizations) employ MSM experiential knowledge to shape HIV responses.
3. Investigate how intersectionality has been operationalized in empirical qualitative studies included in this review.

## Method

Following the Preferred Reporting Items for Systematic Reviews (PRISMA, Page *et al*. 2021), we conducted a systematic review of qualitative and mixed-methods evidence using narrative synthesis (Popay *et al*. 2006) and registered the review protocol in PROSPERO (CRD42024521694). We extracted only the qualitative components of the mixed-methods studies, as this approach aligns with the aim of this systematic review.

### Search strategy

Before conducting the database searches, AHGP reviewed seminal literature to identify key concepts and relevant search terms and drafted the initial search strings. Research librarians at our institution were consulted to refine the strategy and ensure alignment with the aims of this systematic review. Multiple trial searches were conducted in Scopus to assess the adequacy of the search strings in addressing the review questions. To ensure breadth and depth, we employed related terms and constructs aligned with the review’s phenomenon of interest (see Supplementary Material S1).

We conducted our preliminary search across four databases in April 2024: Scopus, MEDLINE, APA PsycINFO, and APA PsycArticles (accessed via Ovid for MEDLINE, APA PsycINFO, and APA PsycArticles). An updated search was conducted in December 2025 to identify additional studies; this search yielded no articles for full-text extraction. Following trial searches across the databases, we assessed the results and finalized the search strings, review questions, and aims.

### Eligibility criteria

Studies were eligible for inclusion if it meets the following criteria: (i) empirical qualitative or mixed-methods studies published in peer-reviewed English-language journals; (ii) studies reporting the experiences of cisgender MSM (We focused on cisgender MSM in this study to ensure that the research accurately reflects their experiences without conflating them with other groups within the LGBTQIA+ community, whose experiences may differ substantially. Including *trans* MSM or non-binary MSM would require distinct methodological considerations to avoid a “one-size-fits-all” approach.), and must be aged 18 years old and above; (iii) studies describing how MSM use their lived experiences to inform HIV health services, including HIV testing, counselling, antiretroviral therapy, pre-exposure prophylaxis (PrEP), post-exposure prophylaxis, clinical trials, research, and community engagement; (iv) studies illustrating how institutions (In some contexts, peer-based and informal settings function as the primary sites of leadership and support.) embed these lived experiences within service provision. Institutional settings included clinics, hospitals, community-based and non-profit organizations, and social contexts such as family and peer networks. To capture intersectionality, we included studies that characterized intersecting forms of marginalization shaping MSM’s experiences, regardless of whether the term *intersectionality* was explicitly used, provided that its core elements were described.

### Screening

All included studies were uploaded to Covidence (https://www.covidence.org), and the deduplication process was conducted using the same software platform. During the initial screening, two reviewers (i.e. AHGP and EW) independently assessed all the articles. AHGP and EW discussed conflicting decisions and eventually reached a consensus on whether to include the references. A similar process was followed during the full-text review. Following the PRISMA guidelines, our full-text review resulted in 40 studies included in the data extraction.

### Extraction

Relevant data were extracted from all included studies. AHGP extracted information on study characteristics (study ID, country, aims, methods, funding, and conflicts of interest), population characteristics (including eligibility criteria, recruitment, and sample size), and analytic content, including MSM’s lived experiences of HIV service provision, how institutions incorporate these experiences into HIV services, and the operationalization of intersectionality.

To ensure consistency and quality, EW independently extracted data from a sample of 10 studies, which were compared with AHGP’s extraction. No significant discrepancies were identified, and AHGP subsequently extracted data from the remaining studies.

### Quality assessment

We used Joanna Briggs Institute (2020) Critical Appraisal Checklist for qualitative research. This checklist evaluates the methodological soundness of the studies included in this systematic review using 10 criteria. These 10 criteria, along with the results of our quality assessment, are shown in Supplementary Material S2. Our quality assessment tool led to the inclusion of all 40 extracted articles for data analysis. EW independently appraised a sample of 10 studies, and the results were compared with those of AHGP. No major discrepancies were identified, and AHGP proceeded to appraise the remaining studies. All 40 studies met the inclusion criteria and were retained for data synthesis, with moderate to high methodological quality.

### Data analysis and synthesis

We uploaded our included studies into NVivo 14 (Lumivero 2023) to assist our data analysis by ensuring systematic organization of our notes, codes, and themes.

Given the diversity of studies included, the various contexts, cultures of MSM, methodologies, and HIV health service provisions, narrative synthesis (Verbeek *et al*. 2012) was deemed appropriate for the qualitative data and our review questions and aims. Narrative synthesis examines patterns across the articles included in this review (Popay *et al*. 2006). In conducting our narrative synthesis, we used reflexive thematic analysis (Braun and Clarke 2019, 2021) to analyze the extracted corpus in the findings and discussion sections of the included studies. This enabled us to identify, analyze, and create themes from the corpus of the studies included in our study. We undertook the following steps: (i) familiarization with qualitative data; (ii) creating codes; (iii) creating initial themes; (iv) reviewing the generated themes; (v) refining the themes; (vi) connecting the corpus into a coherent narrative. Throughout our data analysis and synthesis, we practiced various strategies to achieve trustworthiness and rigor in our data.

The process of familiarizing ourselves with the studies involved thoroughly reading each extracted corpus from all 40 studies. During this process, we maintained reflexive notes, documenting our assumptions, questions, and potential biases that could influence interpretation. Throughout this familiarization phase, we diligently created annotations that captured our inquiries and preliminary observations. Creating codes was initiated by AHGP and had established familiarity with the corpus. Generated codes were annotated in NVivo, employing both latent and semantic coding, yielding descriptive and reflexive codes. Throughout coding, we continually reflected on how our perspectives as researchers might shape interpretation, discussing these reflections within the research team and revising codes and themes accordingly. Following inductive thematic coding, we generated codes without *a priori* assumptions, allowing for nuanced exploration of the corpus regardless of its variation—such as differences in HIV health service provision and the MSM population. Once the corpus had been coded, similar codes were grouped to create initial themes, and annotations were noted. Similar codes were further organized into initial sub-themes and themes, with annotations and memos recording our observations and insights, which helped refine them. We maintained rigor by ensuring that both generated codes and themes aligned with the corpus and by making iterations as needed to ensure they encapsulated it. Each theme generated from our analysis unfolds a narrative that illustrates the experiences of MSM.

### Reflexivity and positionality

Throughout the research process, we employed various reflexive methodologies to ensure we captured the meaning of our qualitative data. Through active notetaking, revisiting the original studies from which the transcripts originated, and group discussions within the research team, we positioned ourselves in relation to the MSM’s narratives in our qualitative data. This, in turn, helped us contextualize the nuances of their experiences. We also acknowledge that our personal values, beliefs, and worldviews have influenced our research project, including the results of this systematic review.

We are a collective of researchers possessing diverse research experiences and backgrounds. AHGP is a first-generation Filipino immigrant researcher, openly cisgender gay man, with lived experience as an HIV community volunteer, researching critical approaches to understanding health inequities among gender and sexually diverse communities. INS is a medical doctor and public health practitioner with previous extensive experience working within HIV service provisions involving key population groups, including MSM in Indonesia. MLR is a non-binary queer researcher located on Ngunnawal country with expertise in sex education, sociology, and queer and feminist theory. BS identifies as an openly queer, *cis*-ish researcher, but whose work comes primarily from a critical health psychology perspective rather than working from the perspective of these lived experiences. We perceive ourselves as both cultural insiders and outsiders in relation to the target population of this systematic review. We recognize the significant influence of worldviews, beliefs, and values on the outcomes of this review. Furthermore, we express our opposition to all forms of stigma and discrimination, irrespective of health condition, gender, sexuality, race, religion, migration status, or other intersecting forms of marginalization.

## Results

### Included studies

**Figure 1:**
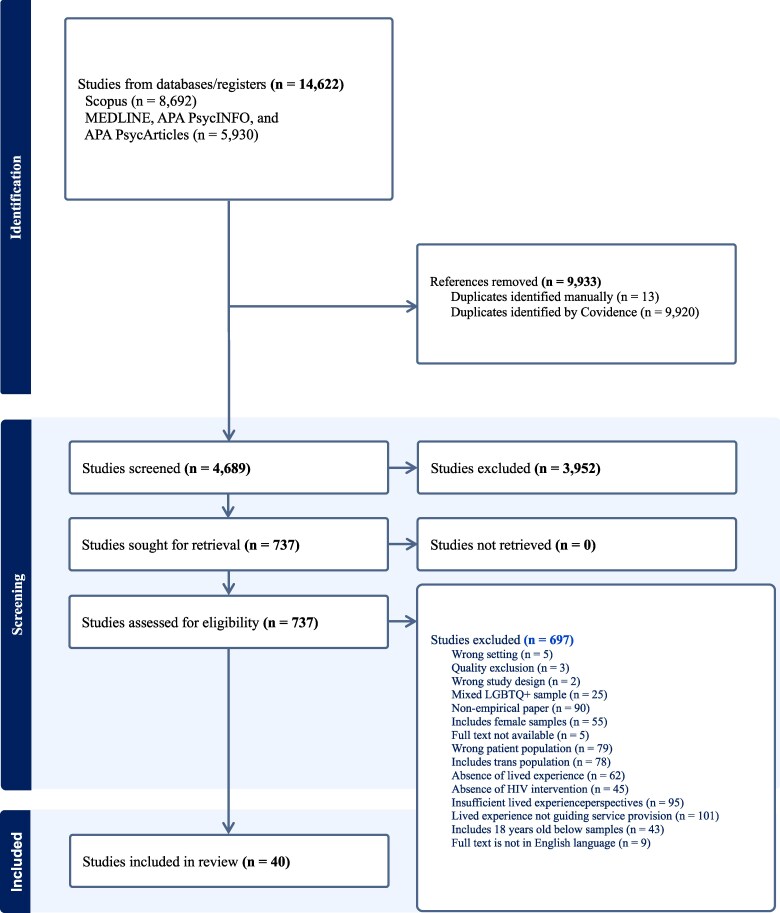
PRISMA flow of included and excluded studies.

Figure 1 shows the PRISMA flow diagram of study selection. We screened 4698 articles, reviewed 737 full texts, and excluded 697, resulting in 40 studies included in the review.

### Characteristics of included studies

Table 1 shows the characteristics of the included studies in this present systematic review.

**Table 1:** Summary of studies included in the narrative synthesis.

Study	Country	Aim of study	Study and analytic design, methodologies	Method of recruitment of participants	Interventions
Jaramillo *et al*. (2024)	USA	To conduct formative research to inform the content, design, and implementation of a scalable, low resource strategy to leverage peer influence to scale up and disseminate PrEP, HIV testing, and BH treatment to LMSM.	Qualitative research; Rapid qualitative analysis; semi-structured interviews	Prior studies, word-of-mouth, and distributing flyers to a community-advisory board (CAB)	HIV intervention
Andrasik *et al*. (2014)	USA	To obtained contextual information regarding documented barriers to HIV clinical trial participation among Black men who have sex with men (MSM), and explored current preventive HIV clinical trial attitudes, beliefs, and perceptions among Black MSM leaders in the United States	Qualitative research; Thematic Analysis); focus groups	Research conference	HIV Clinical Trial
Magnus *et al*. (2014)	USA	To identify factors that may influence engagement and retention of black MSM in HIV research	Qualitative research; in-depth semi-structured key individual interviews; content analysis	Community-based organizations	HIV Research
Garcia *et al*. (2015)	USA	To design a randomized clinical trial (RCT) to evaluate the effectiveness of oral pre-exposure prophylaxis (PrEP) for HIV prevention among Black men who have sex with men (BMSM). The study integrates ethnographic findings into the RCT design to better understand and address the barriers to PrEP adherence in this community.	Mixed-methods; Qualitative: Ethnography; Quantitative: Randomized clinical trial	Community-based organizations	PrEP
Datta *et al*. (2018)	UK	To explore the experiences and views of men who have sex with men (MSM) on attending clinical sexual health services and their preferences regarding service characteristics in the context of the disproportionate burden of STIs experienced by this group. The wider study aim was to develop a risk assessment tool for use in sexual health clinics.	Qualitative research; focus group discussions; thematic analysis	Community-based organizations, newsletters, and social networking sites, and geospatial sociosexual networking	STI Testing
Watson *et al*. (2020)	USA	To describe the formation and implementation of the Black Caucus from the perspective of Black MSM key community stakeholders.	Qualitative research; in-depth interviews; Grounded theory; thematic coding	Community-based organizations, online social networking sites, key informants, and advertising)	HIV Prevention
Quinn *et al*. (2020)	USA	To create the Wisconsin Health Leaders Fellowship program, a yearlong leadership development pilot program for gay and bisexual men of color in Milwaukee, Wisconsin. The Fellowship was developed in response to the lack of leadership positions held by gay and bisexual men of color in agencies providing HIV services in Wisconsin	Formative Evaluation; in-depth interviews; inductive-deductive analysis techniques; content analysis	Clinic patients, telephone, and mail	Leadership
Grieb *et al*. (2021)	USA	To identify community-developed strategies that may enhance community engagement in research with BSMM	Qualitative research; focus groups; Grounded Theory	Community-based organizations, clinics, fliers	HIV Research
Alvarado *et al*. (2021)	Canada and Colombia	To adapt and pilot the accept ≠ ability, appropriateness, and effectiveness of Gay Poz Sex: Finding your own way in Latino MSM.	Mix-methods (Quantitative: Descriptive Statistics; Qualitative: Thematic Analysis; semi-structured one on one interviews)	Clinic patients	HIV intervention
Hussen *et al*. (2022)	USA	To explore social capital among young Black gay and bisexual men living with HIV in Atlanta, Georgia. The researchers focused on understanding the types of social support these men received and how it impacted their lives.	Qualitative research; thematic analysis both code-based approach and case-based approach; in-depth interviews	Youth advisory board	Did not specify
Burchett *et al*. (2022)	USA	To understand how older people living with HIV (OPH) social and health needs can be better served by community programming. We examined the resources (e.g. organizations and programming) that participants were currently accessing; what participants perceived was absent from existing programs; and what they were actively doing in their communities to support the development of programs specifically for OPH.	Qualitative research; focused-group discussions; inductive thematic text analysis	Clinic patients	HIV Engagement
Liboro *et al*. (2021)	Canada	To identify and examine protective factors that fostered resilience to HIV/AIDS based on the insights and lived experiences of racially and ethnically diverse, older GBMSM.	Qualitative research; thematic analysis codebook; interviews	Community-based organizations	Resilience
Kombo *et al*. (2023)	Kenya	To describe a novel community-based program science approach to guide an interdisciplinary research project on HIV self-testing among men who have sex with men in three Kenyan counties. To train MSM in the community to be engaged in HIV research	Qualitative research; community-based program science approach; interpretivist perspective thematic analysis	Community-based organizations	HIV Research
Butts *et al*. (2023)	USA	To qualitatively assess factors affecting Mobile PrEP implementation as perceived by community stakeholders, clients, and program staff.	Qualitative research; thematic content analysis; in-depth interviews	Clinic patients	PrEP
Ramirez-Valles and Brown (2003)	USA	To examine the types of activities, motives, consequences, and deterrents to community involvement among Latino gay men using both community organizations, and Latino gay men.	Qualitative research; No details except mentioned of authors	Community-based organizations, fliers, phone	Community Involvement
Chakrapani *et al*. (2007)	India	To explore the experiences and contexts of stigma and discrimination among HIV-positive and high-risk Kothi-identified men who have sex with men (MSM) in Chennai, India, and ramifications for HIV prevention.	Qualitative research; in-depth semi-structured interviews; Grounded Theory	Community agencies; public sex environments	HIV Prevention
Rhodes *et al*. (2011)	USA	To explore sexual risk among MSM using community-based participatory research (CBPR).	Qualitative research: community-based participatory research; grounded theory; inductive interpretative thematic process	Community-based organizations	Sexual risk
Lorway *et al*. (2014)	India	To describes the engagements of a community of self-identified MSM, most of whom also identified as male sex workers (MSWs), in a long-term iterative and systematic process of knowledge production, reflection, and action.	Qualitative research; community-based participatory research; in-depth qualitative interviews; thematic analysis	Cruising sites	HIV intervention
Chakrapani *et al*. (2012)	India	To explore multi-level factors associated with willingness to participate among MSM in India.	Qualitative research; community-based collaborative approach; focus groups; Key informant interviews; narrative thematic analysis	Community-based organizations	HIV Vaccine Trials
Batist *et al*. (2013)	South Africa	To reach MSM in various townships using community-based social activities and meeting groups. The program was designed to disseminate HIV prevention information and supplies and promote the use of condoms and HIV service uptake.	Mix-methods; Quantitative: Descriptive Statistics; Qualitative: thematic analysis, framework approaches or *a priori*; focus group discussion	Townships	HIV Prevention
Tucker *et al*. (2013)	South Africa	To assess the efficacy of new community-based HIV program (Ukwazana program) and how it promotes effective community engagement, historical feelings of mistrust by Black African MSM of groups tasked to assist them and the harsh social situation defined in large part by township homophobia.	Qualitative research; interviews; thematic analysis	Community-based organizations; townships	HIV Program
Li *et al*. (2017)	China	To explore individual and contextual factors that may influence a linkage to HIV care from the perspective of young HIV-infected men who have sex with men (MSM) in a highly centralized HIV care context of China	Qualitative research; in-depth interviews; thematic analysis	Community-based organizations	HIV Linkage to Care
Tucker *et al*. (2015)	South Africa	To understand how homophobic stigma affects HIV prevention programs for men who have sex with men (MSM) in sub-Saharan Africa, particularly in South Africa. It focuses on the impact of varying levels of homophobic stigma on the implementation and success of community-based HIV prevention programs, such as the Ukwazana program in Cape Town and the Zwakalani program in Johannesburg. The study seeks to highlight the importance of adapting these programs to local contexts and the theoretical implications of sexual binaries on HIV prevention efforts.	Qualitative research; inductive methodology; semi-structured interviews	Townships	HIV Prevention
Buck *et al*. (2017)	El Salvador	To examine the barriers that Salvadoran educators faced in implementing the peer education as designed and adaptations made as a result.	Qualitative research (deductive, codebook); semi-structured interviews	Community-based organizations; community centers	HIV Prevention
Mampane (2018)	South Africa	To explore and describe the experiences of MSM and health care providers (HCPs) regarding the impact of an HIV community engagement program among MSM in a rural community in South Africa.	Qualitative research; in-depth interviews; Thematic analysis	Rural villages	Community Involvement
Adia *et al*. (2019)	The Philippines	To explore the coping strategies to respond to external stress in a collectivistic culture	Qualitative research; interviews; thematic analysis	Clinic patients	HIV Counselling
Ryan *et al*. (2017)	Australia	To explore the acceptability of this service model (PRONTO!) and examine any continued barriers to HIV testing.	Mix methods; in-depth evaluation survey; four focus groups; descriptive and thematic analysis	Clinic patients	HIV Testing
Mutchler *et al*. (2018)	Lebanon	To understand the community dynamics and environmental context of young gay men in Beirut to design effective community-based HIV prevention programs tailored for this group.	Qualitative research; focus groups; peer ethnography; thematic analysis	Community-based organizations, word-of-mouth	HIV Prevention
Hassan *et al*. (2018)	South Africa	To explore the engagement of South African males who have sex with males (MSM) in clinical HIV prevention research. The study focuses on understanding the challenges and strategies for creating safe spaces, addressing stigma and discrimination, and involving community stakeholders to support MSM in the context of HIV prevention.	Qualitative research; in-depth qualitative interviews; thematic analysis	Clinic patients	HIV Engagement
Witzel *et al*. (2018)	UK	To understand the dimensions of acceptability of a potential PrEP service for BMSM aged 18–45 years in London.	Qualitative research; in-depth semi-structured interviews; intersectionality; thematic analysis	Social networking applications, social media, mailing list	PrEP
Sun *et al*. 2019	USA	To describe the needs, assets, and priorities of Black MSM with HIV who live in the Southern United States and identify actions to improve their health using photovoice.	Qualitative research; Photovoice; thematic analysis	Clinic patients	Did not specify
Abubakari *et al*. (2021)	Ghana	This study examined the feasibility and acceptability of an evidence-based HIV prevention program for men who have sex with men (MSM) in Ghana through a participatory approach.	Mix-methods (Quantitative: Descriptive Statistics; Qualitative: focused-group discussions; summative content analysis)	Community-based organizations	HIV Prevention
Mootz *et al*. (2020)	USA	To evaluate the perspectives of MSM with diverse race/ethnicity and age on the acceptability of predictive analytics to determine individual HIV risk and to determine acceptability of having targeted prevention messaging based upon those risk estimates sent directly to the consumer.	Qualitative research; focus group discussions; grounded theory; thematic codebook	Community-based organizations, social media, dating sites, clinics	HIV Prevention
Hassan and Tucker (2021)	South Africa	To examine experiences of engagement in urban MSM-targeted HIV interventions.	Qualitative research; qualitative interviews; thematic analysis	Townships	Community Involvement
Li *et al*. (2020)	China	To expand the knowledge of barriers to good quality of care among MSM living with HIV by adding the views of diverse key stakeholders in two Chinese cities with different HIV care models.	Qualitative research; in-depth interviews; thematic analysis codebook; interviews	Community-based organizations	HIV Care
Jones *et al*. (2022)	USA	To explore the experiences of Black GBMSM working in HIV treatment and prevention, with a focus on understanding the role of their intersectional identities in their work.	Qualitative research; thematic analysis Codebook; secondary analysis from qualitative interviews	Community-advisory board	HIV Prevention
Turpin *et al*. (2025)	USA	To explore these experiences among BSMM in MPowerment models focused on HIV prevention	Qualitative research; in-depth interviews; thematic analysis	Community-based organizations; MPowerment events	HIV intervention
Luo *et al*. (2024)	China	To understand how the standardized patients (SP) experience of conducting unannounced visits shaped their relationship with the MSM community (engagement). To explore the possible impacts of the SP experience on these individuals, as sexual health knowledge and health-seeking behaviors	Qualitative research: in-depth interviews; focus group discussion; inductive codebook	Clinic patients	HIV Stigma Reduction
Hill *et al*. (2023)	USA	To identify the barriers and facilitators to the use of daily oral pre-exposure prophylaxis (PrEP) among young Black gay and bisexual men (YBGBM) in Atlanta. The study sought to understand the structural, interpersonal, and individual determinants of PrEP use, as well as the impact of intersectional stigma on these determinants.	Qualitative research (staged deductive-inductive thematic analysis; in-depth individual interviews	Community-based organizations	PrEP
Reyniers *et al*. (2023)	Burkina Faso, Côte d'Ivoire, Mali, and Togo	To explore the perceptions of West African MSM toward PrEP and their proposed strategies to overcome barriers to PrEP uptake within their communities.	Qualitative research; focus group discussions; semi-structured interviews; Grounded Theory	Community-based organizations	PrEP

Table 2 shows how intersectionality and lived experience leadership have been analytically operationalized and used by the authors of the included studies in this present systematic review.

**Table 2:** Analytic engagement of intersectionality and lived experience leadershi*p.*

Study	Country	Population	Operationalization of intersectionality	Social positions identified in studies	Systems of power or privilege identified in studies^a^	Engagement of intersectionality^b^	Engagement of lived experience leadership
Jaramillo *et al*. (2024)	USA	20 Latino MSM	Multiple identities intersect	Migration, race, gender, sexuality, HIV status	Healthcare institutions, law enforcement authorities	Explicit	Peer leadership
Andrasik *et al*. (2014)	USA	16 MSM	Disparities from systems of power and privilege	SES^c^, poverty, race, HIV status	Healthcare institutions, research institutions	Implicit	Peer leadership
Magnus *et al*. (2014)	USA	Black MSM	Multiple identities intersect; disparities from systems of power and privilege	Substance abuse, ethnicity, sexuality, and cultural diversity	Healthcare institutions, research institutions	Implicit	Peer researchers
Garcia *et al*. (2015)	USA	31 Black MSM	Multiple identities intersect; disparities from systems of power and privilege	SES, race, limited access education, unemployment, sex work, HIV status, sexuality	Religious institutions, families, healthcare institutions, research institutions	Explicit	Peer researchers
Datta *et al*. (2018)	UK	61 MSM	Multiple identities intersect	sexuality, HIV status, race, ethnicity, gender	Healthcare institutions	Explicit	Peer leadership
Watson *et al*. (2020)	USA	Black African American	Multiple identities intersect; disparities from systems of power and privilege	sexuality, race, SES	Research institutions	Explicit	Peer researchers
Quinn *et al*. (2020)	USA	17 MSM of color	Multiple identities intersect; disparities from systems of power and privilege	race, sexuality, gender, HIV status, limited access to education	Healthcare institutions	Explicit	Lived experience governance
Grieb *et al*. (2021)	USA	38 black and sexual minority men	Disparities from systems of power and privilege	HIV status, sexuality, gender	Research institutions	Implicit	Peer researchers
Alvarado *et al*. (2021)	Canada and Colombia	46 MSM living with HIV	Disparities from systems of power and privilege	HIV status, sexual behaviors, substance abuse, mental health status	Healthcare institutions	Implicit	Peer leadership
Hussen *et al*. (2022)	USA	28 young Black gay, bisexual, and other MSM	Multiple identities intersect; disparities from systems of power and privilege	sexuality, race, HIV status, SES, poverty, limited access to education	Religious institutions, families	Explicit	Peer leadership
Burchett *et al*. (2022)	USA	10 gay and bisexual men and other MSM	Multiple identities intersect; disparities from systems of power and privilege	HIV status, older people, race, gender	Healthcare institutions	Explicit	Peer leadership
Liboro *et al*. (2021)	Canada	41 racially and ethnically diverse, older gay and bisexual men and other MSM living with HIV	Multiple identities intersect; disparities from systems of power and privilege	HIV status, sexuality, migration, race, gender	Families, employment organizations	Explicit	Peer leadership
Kombo *et al*. (2023)	Kenya	12 MSM community researchers	Multiple identities intersect; disparities from systems of power and privilege	HIV status, sexuality, SES, social class,	Religious institutions, research institutions	Explicit	Peer researcher
Butts *et al*. (2023)	USA	21 mobile PrEP clients	Multiple identities intersect; disparities from systems of power and privilege	HIV status, race, sexuality, and language	Healthcare institutions	Implicit	Peer leadership
Ramirez-Valles and Brown (2003)	USA	13 Latino gay men	Multiple identities intersect; disparities from systems of power and privilege	HIV status, sexuality, race, ethnicity, poverty, SES	Healthcare institutions	Implicit	Peer leadership
Chakrapani *et al*. (2007)	India	21 participants: 10 HIV-positive kothi-identified MSM, 8 high-risk kothi-identified MSM, and 3 key informants	Multiple identities intersect; disparities from systems of power and privilege	HIV status, sexuality	Healthcare institutions, families, law enforcement authorities,	Explicit	Peer leadership
Rhodes *et al*. (2011)	USA	88 MSM (focus group discussions: *n* = 9)	Multiple identities intersect; disparities from systems of power and privilege	masculinity, race, sexuality, mental health status, poverty, migration, ethnicity	Healthcare institutions, families	Implicit	Peer researcher
Lorway *et al*. (2014)	India	60 MSM	Multiple identities intersect; disparities from systems of power and privilege	HIV status, sexuality, sex work	Healthcare institutions, families	Explicit	Peer leadership
Chakrapani *et al*. (2012)	India	12 focus groups with 68 MSM in Chennai and Mumbai (focus groups: *n* = 12); 14 key informant interviews with MSM community leaders and service providers.	Multiple identities intersect; disparities from systems of power and privilege	HIV status, gender, sexuality, sex work, poverty, masculinity, SES, social class	Healthcare institutions, government institutions	Explicit	Peer leadership
Batist *et al*. (2013)	South Africa	Qualitative: gay black MSM (Focus group discussions: *n* = 36; in-depth interviews: *n* = 5)	Disparities from systems of power and privilege	HIV status, sexuality, mental health status	Healthcare institutions	Implicit	Peer leadership
Tucker *et al*. (2013)	South Africa	18 MSM	Disparities from systems of power and privilege	Sexuality, mental health status,	Healthcare institutions	Implicit	Codesign
Li *et al*. (2017)	China	21 Chinese MSM living with HIV	Disparities from systems of power and privilege	HIV status, mental health status	Healthcare institutions	Implicit	Peer leadership
Tucker *et al*. (2015)	South Africa	5 Zwakalani program facilitators	Multiple identities intersect; disparities from systems of power and privilege	HIV status, sexuality, gender	Healthcare institutions	Implicit	Co-design
Buck *et al*. (2017)	El Salvador	18 MSM	Multiple identities intersect; disparities from systems of power and privilege	HIV, sexuality, gender, poverty, limited access to education,	War violence	Implicit	Peer leadership
Mampane (2018)	South Africa	8 MSM	Multiple identities intersect; disparities from systems of power and privilege	HIV status, poverty, SES, sexuality, gender, mental health status	Healthcare institutions	Implicit	Peer leadership
Adia *et al*. (2019)	The Philippines	7 MSM; 7 key informants	Multiple identities intersect; disparities from systems of power and privilege	HIV status, mental health status, sexuality	Families, healthcare institutions	Implicit	Peer leadership
Ryan *et al*. (2017)	Australia	26 MSM	Disparities from systems of power and privilege	HIV status	Healthcare institutions	Implicit	Peer leadership
Mutchler *et al*. (2018)	Lebanon	10 peer ethnographers and 5 focus groups with 5–7 participants each	Multiple identities intersect; disparities from systems of power and privilege	HIV status, Refugee status, migration, sexuality, race	Law enforcement authorities	Explicit	Peer leadership
Hassan *et al*. (2018)	South Africa	10 gay and bisexual men and other MSM	Multiple identities intersect; disparities from systems of power and privilege	HIV status, sexuality, gender, social class, SES, race	Healthcare institutions	Explicit	Peer leadership
Witzel *et al*. (2018)	UK	25 Black and sexual minority men	Multiple identities intersect; disparities from systems of power and privilege	HIV status, race, sexuality	Healthcare institutions, families	Explicit	Peer consultation
Sun *et al*. (2019)	USA	6 Black MSM living with HIV	Multiple identities intersect; disparities from systems of power and privilege	HIV status, sexuality, race, gender	Families, employment organizations, religious institutions	Explicit	Peer consultation
Abubakari *et al*. (2021)	Ghana	Qualitative (Focus groups): 32 MSM participated in the focus group discussions.	Multiple identities intersect; disparities from systems of power and privilege	HIV status, gender, sexuality, race	Church, Families	Explicit	Peer leadership
Mootz *et al*. (2020)	USA	57 participants (Black =21; White/Non-Latino =16; Latino =15; Other =5) identified as men	multiple identities intersect; disparities from systems of power and privilege	Sex worker, limited access to education, poverty, SES, sexuality, HIV status	Violence, healthcare institutions	Explicit	Peer consultation
Hassan and Tucker (2021)	South Africa	20 MSM	multiple identities intersect; disparities from systems of power and privilege	Mental health status, sexuality, gender, race, HIV status	Violence, healthcare institutions	Explicit	Peer leadership
Li *et al*. (2020)	China	17 Chinese MSM	multiple identities intersect; disparities from systems of power and privilege	HIV, sexuality, mental health status,	Families, healthcare institutions	Explicit	Peer consultation
Jones *et al*. (2022)	USA	19 Black gay and bisexual men and other MSM	multiple identities intersect; disparities from systems of power and privilege	Race, sexuality, HIV status, SES, poverty	Healthcare institutions, employment organizations	Explicit	Peer leadership
Turpin *et al*. (2025)	USA	24 MSM	Multiple identities intersect; disparities from systems of power and privilege	Race, sexuality, SES, mental health status, HIV status	Healthcare institutions	Explicit	Peer leadership
Luo *et al*. (2024)	China	11 Chinese MSM	Disparities from systems of power and privilege	HIV status, sexuality	Healthcare institutions, Research institutions	Implicit	Peer researcher
Hill *et al*. (2023)	USA	23 participants (community-based organization leader *n* = 2; community health outreach workers *n* = 2; clinical PrEP coordinator *n* = 3; public health educator *n* = 1; HIV program administrator *n* = 2; nurse *n* = 1; Clinical PrEP promotion team *n* = 5; PrEP clients *n* = 7)	Multiple identities intersect; disparities from systems of power and privilege	HIV status, race, sexuality, mental health status	Healthcare institutions	Explicit	Peer leadership
Reyniers *et al*. (2023)	Burkina Faso, Côte d'Ivoire, Mali, and Togo	97 MSM with 12 focus group discussions	Multiple identities intersect; disparities from systems of power and privilege	HIV status, sexuality	Healthcare institutions	Implicit	Peer leadership

^a^These institutional dynamics include diverse units, some of which may not be formal institutions, but represent social units that fuel or perpetuate the stigmatization and marginalization of MSM.

^b^We engaged intersectionality in two ways: explicitly, when studies directly used the term intersectionality and examined multiple forms of marginalization and their underlying systems of power. Implicit engagement with intersectionality was identified when studies described intersecting forms of marginalization linked to MSM’s social identities without explicitly using intersectional language. For example, we interpreted implicit intersectionality where studies examined co-occurring stigma experiences and articulated how systems of power shaped these experiences. To ensure analytic consistency, these criteria were predefined and applied systematically across all studies during coding and synthesis.

^c^Socio-economic status (SES).

Although none of our synthesized studies explicitly use the term *lived experience leadership,* we employ this framework to characterize how MSM lead with their communities and draw on their lived experiences to inform various HIV services. We operationalize lived experience leadership when MSM assume roles that influence decision-making, service delivery, or community mobilization based on their personal experiences. We examined characteristics such as initiating or leading community-driven efforts, coordinating peer support, and shaping HIV health services through experiential knowledge (Krulic *et al*. 2022). Our analysis showed that MSM often assumed roles, e.g. peer navigators (Adia *et al*. 2019), in their communities and various HIV health service provisions—often informal ones. It is important to note that these forms of lived experience leadership are not conceptualized as existing in a linear progression, nor do they inherently correspond to increasing degrees of formal power or governance authority, as these may vary across contexts and time.

We have also taken into account how lived experience leadership may overlap with community-engaged HIV services (e.g. Loughhead *et al*. 2024) and how lived experience leadership is diverse, existing across various contexts and initiatives, including in the HIV response (e.g. Pamoso *et al*. 2025a). Instead of setting a minimum criterion of lived experience leadership, we operationalize it by how personal experiences of MSM shape decision-making, service delivery, and community mobilization across various HIV services (Stewart *et al*. 2019). We distinguish lived experience leadership from participation or engagement by emphasizing influence rather than involvement alone; while participation and engagement are vital, they do not necessarily constitute lived experience leadership unless they involve the application of lived experience in substantive influence over decision-making, agendas, or forms of knowledge production within HIV services (Stewart *et al*. 2019). Doing so enables us to capture a diversity of types of lived experience leadership rather than to presume specific definitions. Accordingly, studies that engaged MSM and used their personal experiences to inform various HIV services through outreach, service delivery design, and evaluation were included in our present systematic review. Table 2 operationalizes 40 studies included in the synthesis of lived experience leadership across HIV services: (i) Peer consultations, where MSM provide feedback on services without explicit decision-making delegation; (ii) Peer researchers, where MSM collaborate in the design, or conduct, or dissemination of HIV research as experiential experts; (iii) Codesign, where MSM share power with authorities to design and implement HIV response; (iv) Peer leadership, where MSM initiate and or lead community-based action, advocacy, or services delivery; and (v) Lived experience governance, where MSM hold formal decision-making roles within institutional HIV services structures.

### Theme 1: Navigating the terrains of lived experience leadership

This theme illustrates how MSM embodied lived experience leadership by acting as a support system for peers and communities, and how institutions facilitated spaces for such support (Batist *et al*. 2013). MSM provided assistance ranging from navigating the complexities of HIV health services to offering moral support (Batist *et al*. 2013). Despite these efforts, they encountered barriers to developing a support system and achieving lived experience leadership, including structural, social, personal, and organizational challenges within their community-based organizations (Magnus *et al*. 2014). Nonetheless, our analysis highlights the importance of engaging with their communities, as advocacy and involvement serve as acts of resistance against marginalization (Tucker *et al*. 2013). Meaningful community engagement also requires cultural alignment between HIV health services and the needs of MSM and their communities (Jaramillo *et al*. 2024).

#### Sub-theme 1: Support system

MSM demonstrated lived experience leadership through their interactions with HIV health services. They leveraged personal experience to support peers and communities—acting as peer navigators, connecting others to healthcare providers (Li *et al*. 2017), and promoting safer sexual behaviors (Ramirez-Valles and Brown 2003). As peer workers, MSM helped reduce anxiety around accessing HIV services, which led to improved access to HIV medications (Hassan and Tucker 2021).

Beyond healthcare access, MSM leadership fostered psychosocial support within their communities. By creating opportunities to network and engage with peers (Abubakari *et al*. 2021), they facilitated meaningful social and moral support (Batist *et al*. 2013, Mampane 2018). Such support contributed to the formation of communities characterized as inclusive environments (Adia *et al*. 2019). Several studies underscore the importance of lived experience leadership: it not only enhances access to HIV services but also addresses psychosocial challenges faced by MSM and their communities (Ramirez-Valles and Brown 2003, Garcia *et al*. 2015, Li *et al*. 2017, Adia *et al*. 2019, Burchett *et al*. 2022, Hussen *et al*. 2022, Jaramillo *et al*. 2024).

#### Sub-theme 2: Barriers to engagement

Despite opportunities for MSM to lead within their communities, various challenges limited their engagement, affecting how they provide HIV health services and psychosocial support. Structural factors, such as homophobia (Tucker *et al*. 2013) and extreme violence (Buck *et al*. 2017), discouraged MSM from leadership roles, particularly in conservative contexts (Kombo *et al*. 2023). Some MSM preferred discreet engagement with community-based HIV services to maintain accessibility while avoiding family or community scrutiny (Witzel *et al*. 2018).

At the individual level, some MSM described their involvement as temporary or not aligned with their personal calling (Hussen *et al*. 2022), while others cited limited capacity, such as time constraints (Ramirez-Valles and Brown 2003). MSM with negative views toward their sexuality, gender, or HIV status were less likely to engage in leadership or community activities (Hassan *et al*. 2018, Hussen *et al*. 2022). Such perceptions also influenced service preferences, with some favoring traditional hospitals over community-engaged services to avoid stigma, and engaging with community-based testing only for sensitive issues (Kombo *et al*. 2023). Limited HIV knowledge further hindered community engagement, as misconceptions and lack of awareness affected MSM’s ability to lead peers and participate in community-based services (Reyniers *et al*. 2023).

Institutional barriers within community-based organizations included limited leadership opportunities, insufficient resources, and restrictive organizational climates. Some MSM leaders reported underutilization of skills, role stagnation, or confinement to clerical tasks without decision-making authority (Ramirez-Valles and Brown 2003, Quinn *et al*. 2020, Jones *et al*. 2022). Some organizations perceived MSM as “leaders but not managers” (Quinn *et al*. 2020), highlighting challenges in capacity building (Tucker *et al*. 2013, Andrasik *et al*. 2014). Funding limitations further constrained operations and leadership development (Chakrapani *et al*. 2012, Andrasik *et al*. 2014, Magnus *et al*. 2014, Buck *et al*. 2017). Organizational culture, including inconsistent service delivery, also hindered community engagement (Chakrapani *et al*. 2007, Magnus *et al*. 2014, Li *et al*. 2020, Jones *et al*. 2022, Hill *et al*. 2023).

Despite calls to increase authentic representation of MSM in leadership and decision-making, including queer representatives in heteronormative spaces (Hassan *et al*. 2018) and involving HIV service providers (Butts *et al*. 2023), MSM reported feeling underrepresented, contributing to disengagement (Chakrapani *et al*. 2007, Mutchler *et al*. 2018, Watson *et al*. 2020, Turpin *et al*. 2025). Finally, family obligations and risks related to disclosing sexual, gender, or HIV identities influenced community engagement, with some MSM prioritizing family over community leadership (Chakrapani *et al*. 2012).

#### Sub-theme 3: Fostering meaningful community engagement

Despite the challenges associated with community engagement in HIV services, MSM continued to engage within their communities. Findings suggest that meaningful community engagement operates as a form of resistance against oppression and marginalization (Chakrapani *et al*. 2007, Tucker *et al*. 2013, Hassan *et al*. 2018, Adia *et al*. 2019, Hassan and Tucker 2021, Kombo *et al*. 2023, Luo *et al*. 2024).

To strengthen community engagement, several studies emphasized the importance of cultural alignment between MSM communities and institutions providing HIV health services (Abubakari *et al*. 2021, Garcia *et al*. 2016, Jaramillo *et al*. 2024, 3363; Li *et al*. 2020, Mootz *et al*. 2020, Ryan *et al*. 2017). For example, migrant MSM reported greater motivation to access HIV services when these services incorporated the lived experiences of migrant MSM involved in service delivery, fostering cultural relevance and trust (Jaramillo *et al*. 2024).

Our synthesis highlighted diversity (Tucker *et al*. 2013, Magnus *et al*. 2014, Watson *et al*. 2020, Kombo *et al*. 2023, Jaramillo *et al*. 2024) and technology integration (Garcia *et al*. 2016, Ryan *et al*. 2017, Li *et al*. 2020, Mootz *et al*. 2020, Abubakari *et al*. 2021, Jaramillo *et al*. 2024) as key mechanisms for achieving cultural alignment between MSM communities and HIV service institutions. While technology-enhanced services increased community engagement (Garcia *et al*. 2016, Ryan *et al*. 2017, Li *et al*. 2020, Mootz *et al*. 2020, Abubakari *et al*. 2021, Jaramillo *et al*. 2024), reduced HIV-related stigma (Mootz *et al*. 2020), and promoted inclusivity (Garcia *et al*. 2015), some MSM expressed concerns regarding privacy and reduced human interaction (Mootz *et al*. 2020). Nevertheless, several studies emphasized that ethical and culturally responsive use of technology is essential for community leaders to effectively fulfill their roles and sustain meaningful community engagement within MSM communities.

### Theme 2: Negotiating power and agency

This theme illustrates how MSM encounter stigma ranging from subtle biases to overt discrimination rooted in intersecting social identities, which often discourages community engagement and leadership in HIV services. Despite these constraints, MSM negotiated power and agency through education and advocacy, reducing marginalization while asserting their voices for genuine representation and leadership within organizations and communities.

#### Sub-theme 1: Accounts of (intersectional) oppression

MSM experienced biases from community organizations, healthcare workers, and other MSM (Andrasik *et al*. 2014, Li *et al*. 2020), reflecting a persistent lack of awareness of their struggles (Lorway *et al*. 2014). Such biases generated tension, disrespect, isolation, and neglect within organizations (Lorway *et al*. 2014).

Biases also manifested as tokenism, particularly in lived experience roles, resulting in superficial inclusion and constrained agency (Andrasik *et al*. 2014, Quinn *et al*. 2020, Grieb *et al*. 2021, Jones *et al*. 2022). As one Black American MSM described: “I am looked at as that token guy […] the token Black gay man” (Quinn *et al*. 2020, p. 842). These experiences extended to overt discrimination and racism toward MSM volunteers, advocates, and leaders within healthcare and community settings (Chakrapani *et al*. 2007, 2012, Buck *et al*. 2017, Datta *et al*. 2018, Mampane 2018, Mutchler *et al*. 2018, Luo *et al*. 2024). Reports included breaches of confidentiality, humiliation, and public discrimination following HIV disclosure (Chakrapani *et al*. 2012, Mampane 2018, Li *et al*. 2020). Such experiences fostered anticipated stigma (Chakrapani *et al*. 2012, Luo *et al*. 2024), discouraging engagement in communities and HIV services (Buck *et al*. 2017) and negatively affecting MSM’s well-being and leadership capacity—highlighting the strain of balancing advocacy and leadership roles amid ongoing marginalization and oppression.

Findings identified several interlocking systems of oppression, organized around four intersecting nexuses: HIV diagnosis, sexuality, masculinity, poverty, and race. Each intersects with social identities and broader structural and political contexts experienced by MSM and their communities. Within the HIV nexus, MSM experienced heightened vulnerability not only due to living with or being at risk of HIV, but also through compounded risks related to migration and sexual minority status (Chakrapani *et al*. 2007, Mutchler *et al*. 2018, Liboro *et al*. 2021). These intersecting trajectories included sex work (Datta *et al*. 2018), risky sexual behaviors, homophobia, and social isolation (Chakrapani *et al*. 2007, Tucker *et al*. 2013).

Religious institutions also emerged as powerful sources of stigmatization. Conservative heteronormative values frequently marginalized MSM based on their sexual identities and HIV status (Chakrapani *et al*. 2012, Garcia *et al*. 2015, Sun *et al*. 2019), often reinforcing familial norms incompatible with MSM’s identities (Hussen *et al*. 2022). Religious leaders, in some contexts, hindered MSM from engaging with their communities, limiting opportunities for leadership and outreach (Kombo *et al*. 2023).

Intersectional stigma rooted in racism and homonegativity was also evident (Ramirez-Valles and Brown 2003, Andrasik *et al*. 2014, Garcia *et al*. 2015, Witzel *et al*. 2018, Quinn *et al*. 2020, Hussen *et al*. 2022). Constructions of masculinity intersected with HIV, poverty, immigration, race, and risky sexual behaviors (Rhodes *et al*. 2011). Poverty further intersected with homophobia and HIV (Garcia *et al*. 2015, Hassan and Tucker 2021), including exposure to structural violence (Buck *et al*. 2017).

Although some MSM leaders leveraged intersecting identities to connect more effectively with their communities, these identities rendered them vulnerable to ongoing, compounded oppression (Jones *et al*. 2022). For example, Black MSM were often employed as HIV advocates because their intersecting identities facilitated community engagement; however, these identities also exposed them to persistent marginalization (Jones *et al*. 2022). MSM frequently experienced multiple vulnerabilities arising from overlapping stigmatized identities (Mootz *et al*. 2020). Navigating HIV health services—particularly issues related to HIV status disclosure—was shaped by heteronormative values surrounding marriage, filial piety, and gender expectations (Li *et al*. 2020). Intersectional stigma further deterred engagement with HIV health services (Reyniers *et al*. 2023), with MSM encountering multiple, intersecting barriers across service settings (Luo *et al*. 2024). For some MSM, historical trauma and generational distance from the early HIV epidemic further constrained community engagement with HIV health services (Butts *et al*. 2023, Houang *et al*. 2023).

#### Sub-theme 2: Demanding power

Despite pervasive stigma, MSM reclaim agency through awareness and education, enabling them to tackle oppression (Chakrapani *et al*. 2007). Education supports access to HIV services (Li *et al*. 2017, Luo *et al*. 2024) and fosters liberation from stigma (Alvarado *et al*. 2021, Liboro *et al*. 2021, Luo *et al*. 2024, Turpin *et al*. 2025). Moreover, meaningful reform requires critically examining hierarchical health systems and demanding genuine involvement (Watson *et al*. 2020). As one MSM asserted: “Don’t let them make a seat for you at the table. You demand your seat at the table” (Quinn *et al*. 2020, p. 844). Reclaiming agency is central to MSM leadership: liberation from oppression enables MSM to restore power, advocate effectively, and lead HIV health service responses within their communities.

HIV initiatives led by lived experience may advance social justice and collective action among MSM and their communities (Abubakari *et al*. 2021). Community engagement addressed intersectional stigma linked to HIV, homophobia, and poverty (Mampane 2018). MSM-led communities created safe spaces where individuals could discuss intersectional concerns and express their sexualities openly (Turpin *et al*. 2025). Evidence also showed that MSM formed meaningful relationships to mitigate the social and clinical impacts of HIV, strengthening collective resilience (Liboro *et al*. 2021). Intersectionality not only elucidates how systems of power and privilege structure marginalization but also provides a framework through which MSM can reclaim power when recognized as experts and leaders. Social and sexual networks equipped MSM with tools to navigate intersecting systems of oppression (Garcia *et al*. 2015). Spiritual coping strategies further enabled MSM to confront challenges related to HIV, including disclosure within heteronormative and patriarchal contexts (Adia *et al*. 2019). In conservative societies, spiritual and religious coping allowed MSM to build community initiatives without disclosing their HIV serostatus (Adia *et al*. 2019).

To address social and racial segregation produced by intersectional stigma, HIV institutions must prioritize community awareness that centers the visibility and representation of MSM and their lived experiences (Hassan *et al*. 2018). Such representation fosters social capital among MSM, helping mitigate the effects of intersecting oppressions (Hussen *et al*. 2022) and supporting the integration of intersecting identities into HIV interventions (Watson *et al*. 2020).

#### Sub-theme 3: Construction of lived experience leadership identity

MSM who advocate for their communities draw on their personal experiences of oppression, motivating social change and dedication to social justice. Beyond contributing to institutions through HIV health services, MSM experience personal growth and development through community leadership.

MSM cultivate self-efficacy and self-esteem by advocating for others (Ramirez-Valles and Brown 2003, Batist *et al*. 2013), reporting a more positive sense of self (Ramirez-Valles and Brown 2003). Sharing lived experiences fosters agency in navigating HIV-related challenges (Adia *et al*. 2019), promoting health-seeking behaviors (Luo *et al*. 2024) and ownership of sexual health (Li *et al*. 2017), while demonstrating resilience (Datta *et al*. 2018). Personal growth accompanies leadership roles, with MSM gaining maturity and understanding through community engagement with diverse community members (Ramirez-Valles and Brown 2003, Tucker *et al*. 2013, Adia *et al*. 2019, Burchett *et al*. 2022, Jones *et al*. 2022). Leadership provides affirmation (Tucker *et al*. 2015) and supports aging processes (Burchett *et al*. 2022), facilitates collective coping strategies for HIV stigma (Ramirez-Valles and Brown 2003, Adia *et al*. 2019), and enables acquisition of professional skills (Ramirez-Valles and Brown 2003, Abubakari *et al*. 2021).

MSM cultivate altruism by dedicating time and effort to helping peers navigate HIV-related challenges (Ramirez-Valles and Brown 2003, Luo *et al*. 2024), fostering reciprocity and community formation (Ramirez-Valles and Brown 2003, Sun *et al*. 2019, Grieb *et al*. 2021, Luo *et al*. 2024). MSM often describe peers as “blood brothers” (Adia *et al*. 2019, p. 484), highlighting deep communal bonds: “We are a minority, and so now we have to create our own community whereby we are able to voice all the challenges what we deal with on a daily basis” (Hassan and Tucker 2021, p. 601). This commitment extends beyond institutions, helping communities achieve collective goals (Tucker *et al*. 2013), inspiring others to access HIV services (Jaramillo *et al*. 2024), assume leadership (Quinn *et al*. 2020), and represent the community (Watson *et al*. 2020). Despite leadership challenges, MSM maintain their roles as advocates and volunteers, embodying empowerment and critical consciousness (Jones *et al*. 2022).

### Theme 3: Humanization of HIV

This theme illustrates that HIV extends beyond its physical dimensions, constituting a profoundly personal journey for MSM. Their experiences as leaders, advocates, and volunteers interweave with their narratives, revealing a frequent lack of recognition as individuals and being perceived primarily through their HIV status. Sharing experiences within their communities builds trust, supporting MSM leadership in HIV health services.

#### Sub-theme 1: We are not only about HIV

MSM engaging in HIV services are often viewed solely through the lens of HIV risk and status (Alvarado *et al*. 2021). Services tend to prioritize prevention while neglecting social support and community aspects. Some healthcare workers create meaningful connections with MSM (Hill *et al*. 2023), yet MSM recommend care that is not too medicalized and emphasizes humanizing the person. One MSM reflected: “When one has become HIV-positive, should they no longer have sexual feelings? We are also human beings; why is this not discussed by doctors?” (Chakrapani *et al*. 2007). Person-centered community engagement enhances MSM experiences and informs leadership within their communities (Ryan *et al*. 2017).

MSM described navigating HIV services as a personal and cathartic experience, sharing stories of stigma and identity struggles to foster empathy and collective understanding (Lorway *et al*. 2014). Older MSM reflected on surviving early waves of the HIV epidemic, often experiencing guilt or using religion to cope, providing valuable guidance and advocacy lessons for current MSM leaders (Liboro *et al*. 2021). These reflexive recollections from older MSM about the past serves as a powerful tool and resource for community guidance, advocacy, and transformation, demonstrating how current MSM leaders, advocates, and volunteers can inform the current HIV health services through the personal narratives of other MSM.

Trust is crucial in influencing MSM community engagement and lived experience leadership. MSM feel safer when healthcare providers, peers, and leaders share similar social identities, enhancing alignment of services with their preferences and needs (Butts *et al*. 2023, Reyniers *et al*. 2023, Jaramillo *et al*. 2024). Peer interactions foster openness: “When I hear you speaking, I feel that you and I are the same” (Lorway *et al*. 2014, p. 82). Trust cultivates safe spaces and havens (Abubakari *et al*. 2021, Burchett *et al*. 2022), fostering meaningful relationships with healthcare providers and communities (Hill *et al*. 2023). Nurtured trust improves HIV service uptake and outcomes, creating personalized care (Li *et al*. 2017, Mampane 2018, Witzel *et al*. 2018, Mootz *et al*. 2020, Luo *et al*. 2024).

## Discussion

Our systematic review highlights the evolution of community engagement into lived experience leadership among MSM, enacted through both informal and formal roles. Some MSM emerge organically as community advocates and initiators of social change (Hassan and Tucker 2021), while others are formally appointed to inform HIV health services (Quinn *et al*. 2020). Recognizing these distinctions deepens understanding of lived experience leadership in HIV service contexts.

Our synthesis shows how MSM and their organizations form communities aligned with broader traditions of community-engaged HIV responses, where collective agency fosters safe and inclusive care (e.g. Jaramillo *et al*. 2024). However, in some cultural contexts (e.g. Kenya), MSM may avoid conventional community-based initiatives due to stigma and heteronormative bias (Hassan *et al*. 2018, Kombo *et al*. 2023), instead preferring general hospital or clinic settings to protect privacy (Inghels *et al*. 2022, Kombo *et al*. 2023). This underscores the importance of spatial, cultural, and ecological contexts in shaping access to HIV services. Community-engaged HIV responses grounded in lived experience leadership must account for these preferences to ensure meaningful engagement and effective service delivery (Ayala *et al*. 2021).

Our review highlights how gender and sexuality shape how MSM’s access to HIV services (Chakrapani *et al*. 2007). In some non-Western cultures, MSM may describe sexual behavior rather than identity, with masculinity central to relationships—one partner often seen as dominant/masculine, the other effeminate/submissive (Chakrapani *et al*. 2012). These constructions are critical for designing HIV responses, as gender roles influence service-seeking behaviors (Rhodes *et al*. 2011).

At the systems level, our findings corroborate enduring barriers to meaningful community engagement and lived experience leadership among MSM in HIV health services. Similar to mental health consumers’ struggles in achieving authentic representation in systems, policy, education, and service delivery (Scholz *et al*. 2024a, 2024b), MSM face tokenism and power imbalances that hinder representation and leadership in their communities and organizations.

Lived experience leadership does not exist in a vacuum—stakeholders’ perspectives are crucial for an authentic community-engaged HIV response. To characterize such processes, we employ lived experience leadership as a theoretical and practical anchor to elucidate how meaningful community-engaged HIV services are realized among MSM and their communities. Although the term was not explicitly used in included studies, our synthesis shows how this lens illuminates mechanisms that enable or constrain community-engaged HIV services among MSM. Stakeholders held differing views, with some positioning MSM as leaders but not managers due to perceived limits in managerial capacity (Quinn *et al*. 2020). Although lived experience leadership aims for equitable collaboration (Scholz 2022), MSM are not consistently recognized as leaders capable of sustaining lived experience-led HIV services. This reflects critical gaps in health systems and infrastructure, where a lack of capacity building and leadership opportunities hinders MSM and their organizations from fully embodying lived experience leadership.

Our findings suggest MSM exhibit leadership attributes, demonstrating altruism toward others and personal growth when embracing lived experience leadership (Grieb *et al*. 2021, Jones *et al*. 2022). These attributes connect with their intersectional identities; despite marginalization from overlapping stigmas, MSM take “a source of pride and used their social positions to support” others, showing “intersectional resilience” through leadership (Quinn *et al*. 2022, p. 410). This contributes to understanding how lived experience leadership intersects with marginalized identities (Scholz *et al*. 2024a, 2024b) and how MSM reconcile past oppression by giving back to their communities (Quinn *et al*. 2022).

Viewing HIV health services through an intersectional lens helped us understand the interlocking systems of oppression faced by MSM and how intersectionality can foster collective action and social justice. These findings align with studies showing oppression arising from multiple stigmatized identities (Pamoso *et al*. 2025b). Intersectionality can dismantle power structures fueling marginalization, and when institutions acknowledge these systemic injustices, MSM and their communities are more likely to engage safely as advocates, volunteers, and leaders. Furthermore, although the term intersectionality was explicitly used in some of the reviewed studies (e.g. Garcia *et al*. 2015, Abubakari *et al*. 2021), referencing intersectionality does not ensure meaningful engagement with its theoretical foundations. Bauer *et al*. (2021) found that researchers often engage superficially with intersectionality, treating it as a trend (Davis 2008) rather than applying its core principles (Bowleg 2012). Our analysis shows that some studies identify elements of interlocking oppression even without explicitly using the term.

### Implications

Our synthesis highlights two critical areas for HIV responses. First, advocating lived experience leadership—including its complexities—is crucial for transforming HIV health services for MSM. Second, authentic community engagement of MSM in HIV responses is needed. Achieving Ending AIDS by 2030 will likely require HIV-related institutions to embrace lived experience leadership to ensure communities are meaningfully represented.

Implementing lived experience leadership through an intersectional framework in HIV services, policy, education, and evaluation can contribute to more authentic involvement and representation of MSM and their communities. Evidence shows services rooted in MSM lived experiences improve uptake (Hassan and Tucker 2021), and “intersectional identities foster resilience and collective action” (Quinn *et al*. 2022, p. 410). This approach benefits institutions by informing culturally sensitive policies and interventions, fosters long-term community support, and helps redress power imbalances, enhancing representation across diverse MSM populations. In some contexts (e.g. Kenya), MSM avoid community-based HIV services to prevent stigma or unwanted affiliation (Kombo *et al*. 2023). This raises a theoretical question: can lived experience leadership occur when individuals must conceal their identity? Their inability to engage openly limits leadership opportunities, highlighting the need for future research on how structural and cultural barriers shape the visibility and enactment of lived experience leadership.

While past research has uncovered how communities were consulted to improve HIV health services across various countries (Trapence *et al*. 2012), little emphasis has been placed on how lived experience leadership shapes various HIV health services. Findings from this present systematic review extend how experiential expertise of MSM can shape and inform HIV services. Beyond MSM, expanding lived experience leadership through an intersectional framework yields benefits for other key populations with multiple overlapping risks of stigma and discrimination (e.g. sex workers). Using this approach enables the identification of systems of power that fuel oppression and marginalization, as well as potential elements that promote collective action and social justice among other key populations.

Our review underscores the importance of lived experience leadership in HIV services, but implementation depends on health systems’ readiness. Different countries have varying capacities to support MSM’s community engagement, highlighting the need for reforms in policies and infrastructures to embed lived experience leadership. These insights can guide nuanced approaches and strengthen meaningful community engagement in HIV responses. Below, we recommend the following action items for various stakeholders, policy makers, and funding institutions in various HIV responses:

1. Community organizations, including non-governmental organizations involved in HIV responses, should recognize and institutionalize both formal and informal forms of lived experience leadership within HIV services. This requires embedding lived experience leadership within organizational decision-making, policy development, governance, and service evaluation, rather than limiting involvement to advisory or consultative roles. Clearly defining roles, responsibilities, and governance authority for lived experience leaders—particularly MSM—at an organizational level can support the meaningful integration of lived experience leadership within health promotion systems. For example, in HIV service delivery, the experiential expertise of MSM living with HIV can be embedded across governance, peer leadership, and service delivery roles, enabling their lived experience to strengthen community reach, trust-building, and adaptive responses across the HIV care continuum.
2. Various stakeholders across the HIV care continuum and health services—including, but not limited to, key populations (i.e. MSM), policymakers, and health care providers—should collaboratively codesign a lived experience leadership framework to integrate and operationalize it within the HIV care continuum and services. Such codesign processes should be treated as system-level governance strategies rather than a one-time consultation. Organizations must ensure that codesigned framework(s) will be implemented across all facets HIV care continuum and services.
3. Policy makers should develop, revisit, and strengthen policies and legislation pertaining to the HIV care continuum and health services to embed lived experience leadership frameworks, ensuring that lived experience leadership is sustainably implemented and institutionally supported across health systems. Organizations and policy makers must evaluate the existing models of HIV services through cost effective analysis and evaluate whether embedding lived experience leadership in HIV services improves uptake and retention of MSM accessing various HIV services.

### Limitations and future recommendations

Our review has limitations. While we examined MSM perspectives on lived experience leadership across HIV services, we did not analyze differences across specific services (e.g. testing, counseling, PrEP, and ART) or their interaction with health systems and policies, including countries that do not recognize MSM because of discriminatory laws and attitudes. We focused on general perceptions of access rather than service-specific nuances. Limiting the review to English-language empirical studies may have excluded relevant gray literature and non-English research. Furthermore, the way that power operates across the levels of lived experience leadership in our included studies is not often made explicit.

Future studies should examine how MSM are perceived within specific HIV services in country contexts with equivalent approaches to inclusion of MSM, and how institutional and country contexts shape the implementation of intersectionality-informed lived experience leadership, including how power and governance operate across different levels of lived experience leadership. Such work is essential for understanding how MSM leadership is recognized, supported, and integrated within service delivery and how communities navigate engagement and representation.

Researchers should also explore integrating HIV services with other health services, such as mental health. MSM often face syndemic conditions (Ferlatte *et al*. 2018, Smith *et al*. 2022, Pamoso *et al*. 2025a), where multiple health issues co-occur (Singer *et al*. 2017). Understanding these intersections can guide interventions that address both HIV and other pressing health needs of these men and their communities.

### Conclusion

This systematic review synthesizes MSM perspectives on using their lived experiences to inform HIV health services and how these experiences translate into lived experience leadership. Both informal and formal leadership roles emerged, highlighting how MSM construct communities that shape engagement in HIV services. Culture, sexuality, gender, and structural conditions influence this leadership and continue to constrain meaningful community engagement and representation. Intersectionality reveals interlocking systems of oppression while also enabling collective action toward more equitable HIV services. Realizing “Ending AIDS by 2030” requires systematically involving MSM in service delivery, policy, education, and evaluation through lived experience leadership guided by intersectionality, addressing power imbalances, and fostering inclusive, equitable health outcomes.

## Supplementary Material

daag084_Supplementary_Data

## Data Availability

The data and materials are available from the corresponding author upon reasonable request.

## References

[daag084-B1] Abubakari GM, Nelson LE, Ogunbajo A et al Implementation and evaluation of a culturally grounded group-based HIV prevention programme for men who have sex with men in Ghana. Glob Public Health 2021;16:1028–45. 10.1080/17441692.2020.1832555 33050773 PMC8728790

[daag084-B2] Adia AC, Ng MJ, Quilantang MI et al Collective coping strategies for HIV-related challenges among men who have sex with men in Manila, Philippines. AIDS Educ Prev 2019;31:479–90. 10.1521/aeap.2019.31.5.479 31550192

[daag084-B3] Alvarado BE, Martinez-Cajas JL, Mueses HF et al Adaptation and pilot evaluation of an intervention addressing the sexual health needs of gay men living with HIV infection in Colombia. Am J Mens Health 2021;15:1557988321989916. 10.1177/1557988321989916 33511900 PMC7871071

[daag084-B4] Andrasik MP, Chandler C, Powell B et al Bridging the divide: HIV prevention research and black men who have sex with men. Am J Public Health 2014;104:708–14. 10.2105/AJPH.2013.301653 24524520 PMC4025717

[daag084-B5] Ayala G, Sprague L, van der Merwe LL-A et al Peer- and community-led responses to HIV: a scoping review. PLoS One 2021;16:e0260555. 10.1371/journal.pone.0260555 34852001 PMC8635382

[daag084-B6] Batist E, Brown B, Scheibe A et al Outcomes of a community-based HIV-prevention pilot programme for township men who have sex with men in Cape Town, South Africa. J Int AIDS Soc 2013;16:18754. 10.7448/IAS.16.4.18754 24321116 PMC3852355

[daag084-B7] Bauer GR, Churchill SM, Mahendran M et al Intersectionality in quantitative research: a systematic review of its emergence and applications of theory and methods. SSM—Popul Health 2021;14:100798. 10.1016/j.ssmph.2021.100798 33997247 PMC8095182

[daag084-B8] Bowleg L . When black + lesbian + woman ≠ black lesbian woman: the methodological challenges of qualitative and quantitative intersectionality research. Sex Roles 2008;59:312–25. 10.1007/s11199-008-9400-z

[daag084-B9] Bowleg L . The problem with the phrase women and minorities: intersectionality—an important theoretical framework for public health. Am J Public Health 2012;102:1267–73. 10.2105/ajph.2012.300750 22594719 PMC3477987

[daag084-B10] Bowleg L, Malekzadeh A, Mbaba M et al Ending the HIV epidemic for all, not just some: structural racism as a fundamental but overlooked social-structural determinant of the US HIV epidemic. Curr Opin HIV AIDS 2022;17:40–5. 10.1097/COH.0000000000000724 35102051 PMC9109814

[daag084-B11] Braun V, Clarke V. Reflecting on reflexive thematic analysis. Qual Res Sport Exerc Health 2019;11:589–97. 10.1080/2159676X.2019.1628806

[daag084-B12] Braun V, Clarke V. One size fits all? What counts as quality practice in (reflexive) thematic analysis? Qual Res Psychol 2021;18:328–52. 10.1080/14780887.2020.1769238

[daag084-B13] Buck M, Dickson-Gomez J, Bodnar G. Combination HIV prevention strategy implementation in El Salvador: perceived barriers and adaptations reported by outreach peer educators and supervisors. Glob Qual Nurs Res 2017;4:2333393617703198. 10.1177/2333393617703198 28462359 PMC5406143

[daag084-B14] Burchett CO, Johnson SM, Ryann F et al Using focus group feedback to identify patient-centered initiatives for older persons with HIV. Clin Gerontol 2022;45:661–72. 10.1080/07317115.2020.1769245 32497461 PMC8084408

[daag084-B15] Butts SA, Young B, Blackmon J et al Addressing disparities in pre-exposure prophylaxis (PrEP) access: implementing a community-centered mobile PrEP program in South Florida. BMC Health Serv Res 2023;23:1311. 10.1186/s12913-023-10277-1 38012701 PMC10683210

[daag084-B16] Chakrapani V, Newman PA, Shunmugam M et al Structural violence against kothi-identified men who have sex with men in Chennai, India: a qualitative investigation. AIDS Educ Prev 2007;19:346–64. 10.1521/aeap.2007.19.4.346 17685847

[daag084-B17] Chakrapani V, Newman PA, Singhal N et al Willingness to participate in HIV vaccine trials among men who have sex with men in Chennai and Mumbai, India: a social ecological approach. PLoS One 2012;7:e51080. 10.1371/journal.pone.0051080 23226560 PMC3514227

[daag084-B18] Collins PH . Black Feminist Thought: Knowledge, Consciousness, and the Politics of Empowerment, 2nd ed. New York: Routledge, 2000. 10.4324/9780203900055

[daag084-B19] Coren F, Brown MK, Ikeda DJ et al Beyond tokenism in quality management policy and programming: moving from participation to meaningful involvement of people with HIV in New York State. Int J Qual Health Care 2021;33:1–9. 10.1093/intqhc/mzab004 33415331

[daag084-B20] Crenshaw K . Demarginalizing the intersection of race and sex: a black feminist critique of antidiscrimination doctrine, feminist theory and antiracist politics. In: Feminist Legal Theories, Vol. 1989. Chicago: University of Chicago Legal Forum, 1989, 57–80. https://chicagounbound.uchicago.edu/uclf/vol1989/iss1/8

[daag084-B21] Dale S, Ayala G, Logie C et al Addressing HIV-related intersectional stigma and discrimination to improve public health outcomes. Am J Public Health 2022;112:S335–7. 10.2105/AJPH.2022.306738 35763724 PMC9241474

[daag084-B22] Datta J, Reid D, Hughes G et al Places and people: the perceptions of men who have sex with men concerning STI testing: a qualitative study. Sex Transm Infect 2018;94:46–50. 10.1136/sextrans-2016-052983 28778980 PMC5800331

[daag084-B23] Davis K . Intersectionality as buzzword: a sociology of science perspective on what makes a feminist theory successful. Fem Theory 2008;9:67–85. 10.1177/1464700108086364

[daag084-B24] Ferlatte O, Salway T, Trussler T et al Combining intersectionality and syndemic theory to advance understandings of health inequities among Canadian gay, bisexual and other men who have sex with men. Crit Public Health 2018;28:509–21. 10.1080/09581596.2017.1380298

[daag084-B25] Garcia J, Colson PW, Parker C et al Passing the baton: community-based ethnography to design a randomized clinical trial on the effectiveness of oral pre-exposure prophylaxis for HIV prevention among black men who have sex with men. Contemp Clin Trials 2015;45:244–51. 10.1016/j.cct.2015.10.005 26476286 PMC4674295

[daag084-B26] Garcia J, Parker C, Parker RG et al Psychosocial implications of homophobia and HIV stigma in social support networks. Health Educ Behav 2016;43:217–25. 10.1177/1090198115599398 27037286 PMC4973624

[daag084-B27] Grieb SM, Jackman KM, Tilchin C et al Recommendations from black sexual minority men: building trust to improve engagement and impact of HIV/STI research. Health Promot Pract 2021;22:395–403. 10.1177/1524839920947679 32814464

[daag084-B28] Happell B, Scholz B. Doing what we can, but knowing our place: being an ally to promote consumer leadership in mental health. Int J Ment Health Nurs 2018;27:440–7. 10.1111/inm.12404 29171920

[daag084-B29] Hassan NR, Swartz L, Kagee A et al There is not a safe space where they can find themselves to be free”: (Un)safe spaces and the promotion of queer visibilities among township males who have sex with males (MSM) in Cape Town, South Africa. Health Place 2018;49:93–100. 10.1016/j.healthplace.2017.11.010 29227887

[daag084-B30] Hassan NR, Tucker A. We have to create our own community”: addressing HIV/AIDS among men who have sex with men (MSM) in the neuropolis. Transactions of the Institute of British Geographers 2021;46:598–611. 10.1111/tran.12433

[daag084-B31] Hill M, Smith J, Elimam D et al Ending the HIV epidemic PrEP equity recommendations from a rapid ethnographic assessment of multilevel PrEP use determinants among young black gay and bisexual men in Atlanta, GA. PLoS One 2023;18:e0283764. 10.1371/journal.pone.0283764 36996143 PMC10062590

[daag084-B32] Houang ST, Kafka JM, Choi SK et al Co-occurring epidemic conditions among Southern U.S. black men who have sex with men in an online Ehealth intervention. AIDS Behav 2023;27:641–50. 10.1007/s10461-022-03799-y 35986818 PMC9391640

[daag084-B33] Hussen SA, Drumhiller K, Emerenini S et al Understanding social capital among young black gay and bisexual men living with HIV: a qualitative exploration. Cult Health Sex 2022;24:1498–513. 10.1080/13691058.2021.1974561 34506268 PMC12904984

[daag084-B34] Inghels M, Kouassi AK, Niangoran S et al Preferences and access to community-based HIV testing sites among men who have sex with men (MSM) in Côte d’Ivoire. BMJ Open 2022;12:e052536. 10.1136/bmjopen-2021-052536 PMC9237902 35760538

[daag084-B35] Jaramillo J, Nequiel R, Pranusha A et al Peer ambassador stories: formative qualitative research to enhance the reach of PrEP, HIV testing, and behavioral health treatments to LMSM in South Florida. AIDS Care 2024;36:569–79. 10.1080/09540121.2023.2287736 38157344 PMC10932813

[daag084-B36] Joanna Briggs Institute . 2020 Checklist for qualitative research: critical appraisal tools for use in JBI systematic reviews. JBI Global 1:1–7. https://jbi.global/sites/default/files/2019-05/JBI_Critical_Appraisal-Checklist_for_Qualitative_Research2017_0.pdf

[daag084-B37] Jones M, Smith JC, Moore S et al Passion, commitment, and burnout: experiences of black gay men working in HIV/AIDS treatment and prevention in Atlanta, GA. PLoS One 2022;17:e0264680. 10.1371/journal.pone.0264680 35947604 PMC9365128

[daag084-B38] Kombo BK, Thomann M, Musyoki H et al From collaborator to colleague: a community-based program science approach for engaging Kenyan communities of gay, bisexual and other men who have sex with men in HIV research. Crit Public Health 2023;33:660–71. 10.1080/09581596.2023.2260935

[daag084-B39] Krulic T, Brown G, Bourne A. A scoping review of peer navigation programs for people living with HIV: form, function and effects. AIDS Behav 2022;26:4034–54. 10.1007/s10461-022-03729-y 35672548 PMC9640407

[daag084-B40] Li C, Barrington C, Jiang H et al Challenges for accessing and maintaining good quality of HIV care among men who have sex with men living with HIV in China: a qualitative study with key stakeholders. AIDS care 2020;32:119–26. 10.1080/09540121.2020.1739210 PMC7165036 32151139

[daag084-B41] Li H, Wei C, Tucker J et al Barriers and facilitators of linkage to HIV care among HIV-infected young Chinese men who have sex with men: a qualitative study. BMC Health Serv Res 2017;17:214. 10.1186/s12913-017-2158-7 28302106 PMC5356377

[daag084-B42] Liboro RM, Yates TC, Bell S et al Protective factors that foster resilience to HIV/AIDS: insights and lived experiences of older gay, bisexual, and other men who have sex with men. Int J Environ Res Public Health 2021;18:8548. 10.3390/ijerph18168548 34444297 PMC8394869

[daag084-B43] Lorway R, Thompson LH, Lazarus L et al Going beyond the clinic: confronting stigma and discrimination among men who have sex with men in Mysore through community-based participatory research. Crit Public Health 2014;24:73–87. 10.1080/09581596.2013.791386

[daag084-B44] Loughhead M, Hodges E, McIntyre H et al Pathways for strengthening lived experience leadership for transformative systems change: reflections on research and collective change strategies. Health Expect 2024;27:e70048. 10.1111/hex.70048 39361254 PMC11447884

[daag084-B45] Lumivero . (2023). *NVivo (Version 14)*. In Lumivero. www.lumivero.com

[daag084-B46] Luo, D., Zhang, W., Meng, S. et al 2024 Advancing community-engaged research methods: a qualitative study of men who have sex with men community contributions to a Chinese HIV stigma study. Stigma Health 3:1–8. 10.1037/sah0000512 PMC12333934 40785781

[daag084-B47] Magnus M, Franks J, Griffith S et al Engaging, recruiting, and retaining black men who have sex with men in research studies: don't underestimate the importance of staffing—lessons learned from HPTN 061, the BROTHERS study. J Public Health Manag Pract 2014;20:E1–9. 10.1097/phh.0000000000000025 PMC4167976 24406940

[daag084-B48] Mampane JN . The impact of an HIV community engagement programme among men who have sex with men in a rural community in South Africa. Afr J Nurs Midwifery 2018;19:1–14. 10.25159/2520-5293/1759

[daag084-B49] Matlapeng KM, Babatunde GB, Gwelo NB et al Accessing HIV services in Botswana: perspectives of men who have sex with men and other stakeholders. Glob Health Action 2023;16:2262197. 10.1080/16549716.2023.2262197 37830348 PMC10578085

[daag084-B50] Mootz JJ, Evans H, Tocco J et al Acceptability of electronic healthcare predictive analytics for HIV prevention: a qualitative study with men who have sex with men in New York city. mHealth 2020;6:11. 10.21037/mhealth.2019.10.03 32270003 PMC7136656

[daag084-B51] Mutchler MG, McDavitt BW, Tran TN et al This is who we are: building community for HIV prevention with young gay and bisexual men in Beirut, Lebanon. Cult Health Sex 2018;20:690–703. 10.1080/13691058.2017.1371334 28922101 PMC5857392

[daag084-B52] Page MJ, McKenzie JE, Bossuyt PM et al The PRISMA 2020 statement: an updated guideline for reporting systematic reviews. BMJ (Clinical research ed.) 2021;372:n71. 10.1136/bmj.n71 PMC8005924 33782057

[daag084-B53] Pamoso AHG, Rasmussen ML, Sutarsa IN et al The whole is greater than the sum of its parts: integrating syndemics and intersectionality in tackling the HIV and mental health epidemics among Filipino gay and bisexual men. PLOS Ment Health 2025a;2:e0000252. 10.1371/journal.pmen.0000252 41661883 PMC12798630

[daag084-B54] Pamoso AHG, Scholz B, Ferolino A. “Because of HIV, it opened my mind”: intersectional stigma experiences among Filipino gay and bisexual men living with HIV. Qual Health Res 2025b;35:1037–52. 10.1177/10497323241280213 39533824 PMC12202831

[daag084-B55] Popay J, Roberts H, Sowden A, et al Guidance on the conduct of narrative synthesis in systematic reviews. A product from the ESRC methods programme version. Lancaster University, 2006.

[daag084-B56] Quinn KG, Dickson-Gomez J, Pearson B et al Intersectional resilience among black gay, bisexual, and other men who have sex with men, Wisconsin and Ohio, 2019. Am J Public Health 2022;112:S405–12. 10.2105/ajph.2021.306677 35763748 PMC9241476

[daag084-B57] Quinn KG, Wolfe H, Vergeront J. Don’t deny yourself a seat at the table”: supporting the leadership development of MSM of color in HIV services. Health Promot Pract 2020;21:838–48. 10.1177/1524839919850563 31148482

[daag084-B58] Ramirez-Valles J, Brown AU. Latinos’ community involvement in HIV/AIDS: organizational and individual perspectives on volunteering. AIDS Educ Prev 2003;15:90–104. 10.1521/aeap.15.1.5.90.23606 12630602 PMC3674846

[daag084-B59] Reyniers T, Babo SAY, Ouedraogo M et al Strategies to improve PrEP uptake among west African men who have sex with men: a multi-country qualitative study. Front Public Health 2023;11:480–8. 10.3389/fpubh.2023.1165327 PMC10167043 37181706

[daag084-B60] Rhodes SD, Hergenrather KC, Vissman AT et al Boys must be men, and men must have sex with women: a qualitative CBPR study to explore sexual risk among African American, Latino, and White gay men and MSM. Am J Men's Health 2011;5:140–51. 10.1177/1557988310366298 20413391 PMC3299539

[daag084-B61] Ryan KE, Pedrana A, Leitinger D et al Trial and error: evaluating and refining a community model of HIV testing in Australia. BMC Health Serv Res 2017;17:692. 10.1186/s12913-017-2635-z 29017561 PMC5635501

[daag084-B62] Scholz B . We have to set the bar higher: towards consumer leadership, beyond engagement or involvement. Aust Health Rev 2022;46:509–12. 10.1071/AH22022 35508415

[daag084-B63] Scholz B, Kirk L, Warner T et al From a single voice to diversity: reframing ‘representation’ in patient engagement. Qual Health Res 2024a;34:1007–18. 10.1177/10497323231221674 38229426 PMC11487870

[daag084-B64] Scholz B, Stewart S, Pamoso A et al The importance of going beyond consumer or patient involvement to lived experience leadership. Int J Ment Health Nurs 2024b;33:1–4. 10.1111/inm.13282 38131453

[daag084-B65] Singer M, Bulled N, Ostrach B et al Syndemics and the biosocial conception of health. Lancet (London, England) 2017;389:941–50. 10.1016/s0140-6736(17)30003-x 28271845

[daag084-B66] Smith L, Patel V, Md MPH et al Integrating intersectional and syndemic frameworks for ending the US. Am J Public Health 2022;112:S340–3. 10.2105/AJPH.2021.306634 35763739 PMC9241475

[daag084-B67] Stangl AL, Atkins K, Leddy AM et al What do we know about interventions to reduce intersectional stigma and discrimination in the context of HIV? A systematic review. Stigma Health 2023;8:393–408. 10.1037/sah0000414 42016451 PMC13095170

[daag084-B68] Stewart S, Scholz B, Gordon S et al It depends what you mean by leadership’: an analysis of stakeholder perspectives on consumer leadership. Int J Ment Health Nurs 2019;28:339–50. 10.1111/inm.12542 30281898

[daag084-B69] Sun CJ, Nall JL, Rhodes SD. Perceptions of needs, assets, and priorities among black men who have sex with men with HIV: community-driven actions and impacts of a participatory photovoice process. Am J Mens Health 2019;13:1557988318804901. 10.1177/1557988318804901 30296869 PMC6440064

[daag084-B70] Trapence G, Collins C, Avrett S et al From personal survival to public health: community leadership by men who have sex with men in the response to HIV. Lancet (London, England) 2012;380:400–10. 10.1016/S0140-6736(12)60834-4 22819662 PMC3805044

[daag084-B71] Tucker A, de Swardt G, McIntyre J et al How do community-based HIV prevention programmes for men who have sex with men ‘travel’? Lessons from the Ukwazana/Zwakalani journey in South Africa. Cult Health Sex 2015;17:977–89. 10.1080/13691058.2015.1018948 25752360

[daag084-B72] Tucker A, De Swardt G, Struthers H et al Understanding the needs of township men who have sex with men (MSM) health outreach workers: exploring the interplay between volunteer training, social capital and critical consciousness. AIDS Behav 2013;17:S33–42. 10.1007/s10461-012-0287-x 22903420

[daag084-B73] Turpin RE, Camp AD, Mandell CJ et al Black sexual minority men’s experiences in MPowerment interventions: implications for HIV prevention. Int J Behav Med 2025;32:360–70. 10.1007/s12529-024-10275-5 38499962 PMC11563193

[daag084-B74] UNAIDS . *Global AIDS Strategy 2021–2026—End Inequalities. End AIDS*. 2021. https://www.unaids.org/en/resources/documents/2021/2021-2026-global-AIDS-strategy

[daag084-B75] Verbeek J, Ruotsalainen J, Hoving JL. Synthesizing study results in a systematic review. Scand J Work Environ Health 2012;38:282–90. 10.5271/sjweh.3201 22015561

[daag084-B76] Watson CC, Wilton L, Lucas JP et al Development of a black caucus within the HIV prevention trials network (HPTN): representing the perspectives of black men who have sex with men (MSM). Int J Environ Res Public Health 2020;17:871. 10.3390/ijerph17030871 32028553 PMC7037695

[daag084-B77] Witzel TC, Nutland W, Bourne A. What qualities in a potential HIV pre-exposure prophylaxis service are valued by black men who have sex with men in London? A qualitative acceptability study. Int J STD AIDS 2018;29:760–5. 10.1177/0956462418755224 29466919 PMC7614225

