# Supplementary material for: From community engagement to lived experience leadership: a systematic review of HIV services among men who have sex with men
Source: Health Promot Int. 2026 Jul 2;41(4):daag084. doi: 10.1093/heapro/daag084 (PMC13394705; doi:10.1093/heapro/daag084)
Supplement: daag084_Supplementary_Data [file daag084_supplementary_data.zip › Supplementary Material 1.docx]

# **Supplementary Material 1**

# **Database-Specific Search Strings^[[1]](#footnote-1)^**

# SCOPUS

## *Population: Gay Men Or Bisexual Men Or MSM*

TITLE-ABS-KEY(bisexual*) OR

TITLE-ABS-KEY(gay) OR

TITLE-ABS-KEY(homosexual*) OR

TITLE-ABS-KEY("men who have sex with men") OR

TITLE-ABS-KEY("men having sex with men") OR

TITLE-ABS-KEY("sexual W/3 minorit*") OR

TITLE-ABS-KEY("non heterosexual*") OR

TITLE-ABS-KEY(msmw) OR

TITLE-ABS-KEY(msm) OR

TITLE-ABS-KEY("same sex intercourse*")

OR

TITLE-ABS-KEY("LGBT*") OR

TITLE-ABS-KEY("LGB*") OR

TITLE-ABS-KEY("sexual minority") OR

TITLE-ABS-KEY("sexual orientation")

AND

## *Phenomenon: Intersectionality*

(TITLE-ABS-KEY(intersect* OR multipl* OR interlock* OR overlap* OR layer* OR nexus))

AND (TITLE-ABS-KEY("LGBT*" OR "sexual minority"))

OR

## *Phenomenon: Stigma*

TITLE-ABS-KEY(stigma OR discriminat* OR equality OR inequal* OR equity OR inequit* OR oppress* OR racis* OR sexis* OR homophobi* OR transphobi* OR marginaliz* OR prejudice OR stereotyp*)

AND

## *Phenomenon: HIV*

TITLE-ABS-KEY("HIV" OR "AIDS" OR "human immunodeficiency virus" OR "Acquired immune deficiency syndrome")

AND

## *Phenomenon: Lived Experience Leadership*

TITLE-ABS-KEY("consumer involve*" OR "patient involv*" OR "user involv*" OR "peer involv*" OR "carer involv*" OR

"consumer lead*" OR "patient lead*" OR "user lead*" OR "peer lead*" OR "carer lead*" OR

"consumer control*" OR "patient control*" OR "user control*" OR "peer control*" OR "carer control*" OR

"consumer run" OR "patient run" OR "user run" OR "peer run" OR "peer-run" OR "carer run"

OR

TITLE-ABS-KEY("communit* led" OR "communit* led organization*" OR "communit* led engagement*" OR "communit* engagement*" OR

"communit* base*" OR "communit* base* organization*" OR "communit* base* engagement*" OR "communit

OR

TITLE-ABS-KEY("key population led organiz*" OR "key population leader*" OR "key population led engagement*" OR

"key population base*" OR "key population base* organiz*" OR "key population base* engagement*" OR

"key population engage*" OR "key population organiz*")

OR

TITLE-ABS-KEY("key population led response*" OR "key population response*" OR "key population response* organiz*" OR "key population response* engagement*")

# **Ovid (PsycINFO, PsycArticles, and MEDLINE)**

## *Population: Gay and Bisexual*

("bisexual*" OR "gay" OR "homosexual*" OR "men who have sex with men" OR "men having sex with men" OR "sexual W/3 minort*" OR "non heterosexual" OR "msmw" OR "msm" OR "same sex intercourse" OR “sexual and gender minorit*” OR “sexual minority” OR “sexual orientation” OR “LGBT*” OR “LGB*” OR “sexual minorit*” OR gender minorit* OR “male” OR “men” OR “male homosexual*” OR “male bisexual*” OR “queer*” or “same sex attract*”)

AND

## *Phenomenon: Intersectionality*

(intersect* OR multipl* OR interlock* OR overlap* OR layer* OR nexus)

AND/OR

## *Phenomenon: Stigma*

(“stigma” OR “discriminat*” OR “equality” OR “inequal*” OR “equity” OR “inequit*” OR “oppress*” OR “racis*” OR “sexis*” OR “homophobi*” OR “transphobi*” OR “marginaliz*”OR “prejudice” OR “stereotyp*”)

AND

## *Phenomenon: Syndemics, HIV, mental health*

("HIV" OR "AIDS" OR "human immunodeficiency virus" OR "Acquired immune deficiency syndrome")

OR

("mental disorder*" OR "mental illness*" OR "mental condition*" OR "mental health" OR "mental health disorder*" OR "mental health illness*" OR "psychological health disorder*" OR "psychological disorder*" OR "psychological illness*" OR "psychopatholog*" OR "psychiatric disorder*" OR "behavioral health")

AND/OR

## *Phenomenon: Lived Experience Leadership*

("consumer involv*" OR "patient involv*" OR "user involv*" OR "peer involv*" OR "carer involv*" OR "consumer lead*" OR "patient lead*" OR "user lead*" OR "peer lead*" OR "carer lead*"OR "consumer control*" OR "patient control*" OR "user control*" OR "peer control*" OR "carer control*" OR "consumer run" OR "patient run" OR "user run" OR "peer-run" OR "carer run"))

OR

"communit* led" OR "communit* led organization*" OR "communit* led engagement*" OR "communit* engagement*" OR "communit* base*" OR "communit* base* organization*" OR "communit* base* engagement*" OR "communit* organiz*").mp.

OR

("key population led organiz*” OR “key population leader*” OR “key population led engagement*” OR "key population base*” OR “key population base* organiz*” OR “key population base* engagement*” OR “key population engage*” OR “key population* organiz*”)

OR

("key population led response*” OR “key population response*” OR "key population response* OR “key population response* organiz*” OR “key population response* engagement*”))

OR

(“consumer involve*” OR "patient involv*" OR "user involv*" OR "peer involv*" OR "carer involv*" OR "consumer lead*" OR "patient led*" OR "user lead*" OR "peer lead*" OR "carer lead*" OR "consumer control*" OR "patient control*" OR "user control*" OR "peer control" OR "carer control*" OR "consumer run" OR "patient run" OR "user run" OR "peer-run" OR "carer run" OR "communit* led" OR "communit* led organization*" OR "communit* led engagement*" OR "communit* engagement*" OR "communit* base*" OR "communit* base* organization*" OR "communit* base* engagement*" OR "communit* organiz" OR "key population led organiz*" OR "key population leader*" OR "key population led engagment*" OR "key population base*" OR ""key population base* organiz*" OR "key population base* engagement*" OR "key population engage*" OR "key population* organiz*" OR "key population led response*" OR "key population response*" OR "Key population response* organiz* OR "key population response* engagement*"

1. We adapted some of the search terms from the past works of [Ouafik et al., 2022](https://doi.org/10.1016/j.socscimed.2022.115162), [Scholz et al., 2017](https://doi.org/10.1111/inm.12266), and [Stangl et al., 2023](https://psycnet.apa.org/doi/10.1037/sah0000414) [↑](#footnote-ref-1)
