# Supplementary material for: From community engagement to lived experience leadership: a systematic review of HIV services among men who have sex with men
Source: Health Promot Int. 2026 Jul 2;41(4):daag084. doi: 10.1093/heapro/daag084 (PMC13394705; doi:10.1093/heapro/daag084)
Supplement: daag084_Supplementary_Data [file daag084_supplementary_data.zip › Supplementary Material 2.docx]

| **Supplementary File 2**  *Quality Assessment* | | | | | | | | | | | | |
| --- | --- | --- | --- | --- | --- | --- | --- | --- | --- | --- | --- | --- |
| Author | Is there congruity between the stated philosophical perspective and the research methodology? | Is there congruity between the research methodology and the research question or objectives? | Is there congruity between the research methodology and the methods used to collect data? | Is there congruity between the research methodology and the representation and analysis of data? | | Is there congruity between the research methodology and the interpretation of results? | Is there a statement locating the researcher culturally or theoretically? | Is the influence of the researcher on the research, and vice versa, addressed? | Are participants, and their voices, adequately represented? | Is the research ethical according to current criteria or, for recent studies, and is there evidence of ethical approval by an appropriate body? | Do the conclusions drawn in the research report flow from the analysis, or interpretation, of the data? |  |
| Jaramillo 2024 | Yes | Yes | Yes | | Yes | Yes | Yes | Yes | Yes | Yes | Yes |  |
| Andrasik 2014 | Yes | Yes | Yes | | Yes | Yes | No | Yes | Yes | Yes | Yes |  |
| Magnus 2014 | Yes | Yes | Yes | | Yes | Yes | Unclear | Unclear | Yes | Yes | Yes |  |
| Garcia 2015 | Yes | Yes | Yes | | Yes | Yes | Yes | Yes | Yes | Yes | Yes |  |
| Datta 2018 | Yes | Yes | Yes | | Yes | Yes | Unclear | Unclear | Yes | Yes | Yes |  |
| Watson 2020 | Yes | Yes | Yes | | Yes | Yes | Unclear | Yes | Yes | Yes | Yes |  |
| Quinn 2020 | Yes | Yes | Yes | | Yes | Yes | No | No | Yes | Unclear | Yes |  |
| Grieb 2021 | Yes | Yes | Yes | | Yes | Yes | Unclear | Unclear | Yes | Yes | Yes |  |
| Alvarado 2021 | Yes | Yes | Yes | | Yes | Yes | Yes | No | Yes | Yes | Yes |  |
| Hussen 2022 | Yes | Yes | Yes | | Yes | Yes | Unclear | Unclear | Yes | Yes | Yes |  |
| Burchett 2022 | Yes | Yes | Yes | | Yes | Yes | Unclear | Unclear | Yes | Yes | Yes |  |
| Liboro 2021 | Yes | Yes | Yes | | Yes | Yes | Unclear | Unclear | Yes | Yes | Yes |  |
| Kombo 2023 | Yes | Yes | Yes | | Yes | Yes | Yes | Yes | Yes | Yes | Yes |  |
| Butts 2023 | Yes | Yes | Yes | | Yes | Yes | Unclear | Unclear | Yes | Yes | Yes |  |
| Ramirez-Valles 2003 | Yes | Yes | Yes | | Yes | Yes | Unclear | Unclear | Yes | Unclear | Yes |  |
| Chakrapani 2007 | Yes | Yes | Yes | | Yes | Yes | Unclear | Unclear | Yes | Yes | Yes |  |
| Rhodes 2011 | Yes | Yes | Yes | | Yes | Yes | Yes | Yes | Yes | Yes | Yes |  |
| Lorway 2014 | Yes | Yes | Yes | | Yes | Yes | Yes | No | Yes | Unclear | Yes |  |
| Chakrapani 2012 | Yes | Yes | Yes | | Yes | Yes | Unclear | Unclear | Yes | Yes | Yes |  |
| Batist 2013 | Yes | Yes | Yes | | Yes | Yes | No | Yes | Yes | Yes | Yes |  |
| Tucker 2013 | Yes | Yes | Yes | | Yes | Yes | Unclear | Unclear | Yes | Unclear | Yes |  |
| Li 2017 | Yes | Yes | Yes | | Yes | Yes | Unclear | Unclear | Yes | Yes | Yes |  |
| Tucker 2015 | Yes | Yes | Yes | | Yes | Yes | Unclear | Unclear | Yes | Yes | Yes |  |
| Buck 2017 | Yes | Yes | Yes | | Yes | Yes | Yes | Yes | Yes | Yes | Yes |  |
| Mampane 2017 | Yes | Yes | Yes | | Yes | Yes | Unclear | Unclear | Yes | Yes | Yes |  |
| Adia 2019 | Yes | Yes | Yes | | Yes | Yes | No | Yes | Yes | Yes | Yes |  |
| Ryan 2017 | Yes | Yes | Yes | | Yes | No | Unclear | Unclear | Yes | Yes | Yes |  |
| Mutchler 2018 | Yes | Yes | Yes | | Yes | Yes | No | Yes | Yes | Yes | Yes |  |
| Hassan 2018 | Yes | Yes | Yes | | Yes | Yes | Unclear | Unclear | Yes | Yes | Yes |  |
| Witzel 2018 | Yes | Yes | Yes | | Yes | Yes | Unclear | Unclear | Yes | Yes | Yes |  |
| Sun 2019 | Yes | Yes | Yes | | Yes | Yes | Unclear | Unclear | Yes | Yes | Yes |  |
| Abubakari 2021 | Yes | Yes | Yes | | Yes | Yes | No | No | Yes | Unclear | Yes |  |
| Mootz 2020 | Yes | Yes | Yes | | Yes | Yes | Unclear | Unclear | Yes | Yes | Yes |  |
| Hassan 2021 | Yes | Yes | Yes | | Yes | Yes | Unclear | Unclear | Yes | Yes | Yes |  |
| Li 2020 | Yes | Yes | Yes | | Yes | Yes | Unclear | Unclear | Yes | Yes | Yes |  |
| Jones 2022 | Yes | Yes | Yes | | Yes | Yes | Unclear | Unclear | Yes | Yes | Yes |  |
| Turpin 2024 | Yes | Yes | Yes | | Yes | Yes | Yes | No | Yes | Yes | Yes |  |
| Luo 2024 | Yes | Yes | Yes | | Yes | Yes | Yes | Yes | Yes | Yes | Yes |  |
| Hill 2023 | Yes | Yes | Yes | | Yes | Yes | Yes | Yes | Yes | Yes | Yes |  |
| Reyniers 2023 | Yes | Yes | Yes | | Yes | Yes | Yes | Unclear | Yes | Yes | Yes |  |
